# Bridging the Human–AI Trust Gap: The Mechanisms Underlying Perceived Efficacy of AI-Powered Psychological Counseling Large Language Models

**DOI:** 10.3390/bs16101914

**Published:** 2026-10-15

**Authors:** Yi Wang, Mingchao Yin, Kun Lv

**Affiliations:** 1 Business School, Ningbo University, Ningbo 315211, China; 2 Merchants’ Guild Economics and Cultural Intelligent Computing Laboratory, Ningbo University, Ningbo 315211, China

**Keywords:** AI-based psychological counseling, perceived efficacy, perceived professional competence, perceived risk, perceived economic accessibility

## Abstract

Artificial intelligence (AI) is transforming the delivery of mental health services, yet public trust in AI-based psychological counseling remains a significant challenge. Drawing on a “technology–context–trust” framework, this study recruited a total of 1000 consumers in China through Credamo and conducted five between-subjects, scenario-based experiments to examine differences in perceived efficacy between LLM-based psychological counseling and human experts and to investigate the mechanisms underlying these differences. Perceived efficacy was the primary outcome of the study and, in Experiment 1B, also served as a mediator linking counseling provider type to downstream market outcomes, including overall attitude, willingness to try, and willingness to pay. The results showed that (1) consumers generally perceived AI as less efficacious than human experts, a difference driven by the parallel mediating effects of perceived professional competence and perceived risk; (2) participants rated the AI-based service as more efficacious in the routine counseling vignette, whereas they rated the human-expert service as more efficacious in the major psychological-disorder intervention vignette; (3) disclosure of technological investment enhanced trust in AI’s capabilities and mitigated negative evaluations; and (4) safety-standard disclosure reduced perceived risk, while higher perceived economic accessibility was associated with a smaller provider-related difference in perceived professional competence. These findings characterize subjective service evaluations among the recruited online consumers in China and do not establish comparative clinical effectiveness. They inform research on consumer trust while highlighting the need for further validation in more diverse samples and actual service settings.

## 1. Introduction

Large language models (LLMs) are reshaping a wide range of emerging industries, including smart healthcare, intelligent education, and virtual services. In recent years, LLMs have become increasingly integrated into the mental health service sector, giving rise to innovative applications such as the Zizai Mental Health Large Model developed by People’s Daily Online and the AI mental health assistant developed by Woebot Health in the United States (hereafter collectively referred to as AI-based psychological counseling LLMs). AI-based psychological counseling LLMs offer several advantages. First, their 24/7 service model overcomes the temporal constraints of traditional psychological counseling. Second, a systematic review and meta-analysis found that AI-based conversational agents can reduce depressive symptoms and psychological distress, suggesting their potential to support mental health care (Li et al., 2023). Finally, compared with human experts, AI-based services have lower marginal costs and can therefore provide more affordable services to a broader population.

Although AI-based mental health applications are being developed across countries, this study is situated in mainland China, where mental health services have increasingly been incorporated into a broader public health and social service system. National policy has emphasized strengthening the mental health service network and improving the coordination of mental health education, psychological assessment, counseling, treatment, and community-based support (Chen et al., 2023). At the same time, research on Chinese adults indicates that help-seeking can be shaped by self-reliance, low perceived need for professional help, affordability concerns, stigma, and reluctance to disclose mental health difficulties (Shi et al., 2020). These institutional and cultural characteristics provide an important context for understanding how consumers evaluate the competence, risk, accessibility, and efficacy of emerging AI-based psychological counseling services.

However, the development of AI-based psychological counseling LLMs has also generated a series of social controversies. In 2024, a conversational chatbot developed by Character.AI attracted global attention following a serious incident in which it allegedly encouraged an American boy with depression to engage in suicidal behavior. Such incidents have intensified public concerns about potential loss of technological control, with people questioning whether AI possesses the ethical judgment and emotional empathy of human experts. These concerns are particularly salient in highly sensitive situations involving suicidal ideation and traumatic experiences, where algorithmic bias or the “black-box” nature of AI decision making may lead to severe consequences (De Freitas & Cohen, 2024). More specifically, because AI may have limitations in interpreting nonverbal cues, adapting dynamically to contextual changes, and exercising ethical autonomy, AI systems may struggle to achieve levels of professional competence and trust comparable to those of human experts (Sedlakova & Trachsel, 2023).

The widespread adoption of AI technology therefore requires an appropriate balance between technological rationality and humanistic care (Rubeis, 2022). Although numerous studies have examined public acceptance of AI in general commercial customer service and basic medical decision-support settings, relatively limited attention has been paid to psychological counseling, a domain characterized by a strong reliance on emotional connection and heightened ethical sensitivity. Existing studies have often conceptualized human–AI trust as a unidimensional and static outcome, overlooking the diversity of consumers’ psychological needs and the dynamic role of intervention strategies in shaping trust. To address this research gap, this study focuses on perceived efficacy as the core dependent variable, defined as consumers’ overall subjective expectation that a psychological counseling service can effectively alleviate their psychological distress and help them achieve their counseling goals. For example, perceived efficacy reflects the extent to which consumers believe that AI can effectively alleviate persistent insomnia or severe workplace anxiety. In addition, perceived professional competence, referring to consumers’ evaluations of AI’s ability and skills to address psychological problems, and perceived risk, referring to concerns about potential harms such as privacy breaches and ethical violations, are introduced as core constructs for examining the mechanisms underlying human–AI trust.

To address the limitations of existing research, this study develops a triadic “technology–context–trust” framework as the overarching organizing logic. The technology dimension captures provider type and observable signals, including technological investment and safety-standard disclosure. The context dimension captures variation in counseling needs and perceived economic accessibility, and the trust dimension captures consumers’ evaluations of perceived professional competence and perceived risk, which ultimately shape perceived efficacy. Specific theoretical perspectives are used as supporting lenses for individual relationships within these three dimensions rather than as separate overarching frameworks. This structure places the analytical emphasis on how technological characteristics, contextual conditions, and trust-related evaluations jointly shape consumers’ perceived efficacy of AI-based psychological counseling services.

Building on this theoretical framework, the present study conducts five progressively structured scenario-based experiments and advances the following core hypotheses. Compared with human experts, consumers exhibit lower perceived efficacy toward AI-based psychological counseling LLMs (Hypothesis 1). We hypothesize that the difference in perceived efficacy varies across counseling-need contexts, favoring the AI-based provider in routine counseling scenarios (Hypothesis 2a) and the human expert in major psychological-disorder intervention scenarios (Hypothesis 2b). In this process, perceived professional competence (Hypothesis 3) and perceived risk (Hypothesis 4) serve as parallel mediators. Furthermore, the study demonstrates that perceived economic accessibility (Hypothesis 5), technological investment disclosure (Hypothesis 6), and safety-standard disclosure (Hypothesis 7) can effectively moderate key pathways and thereby help bridge the human–AI trust gap. This comprehensive examination, encompassing the main effect, boundary conditions, underlying mechanisms, and intervention pathways, ensures a high degree of alignment between the theoretical logic and empirical analysis and provides both theoretical foundations and practical implications for building trust in AI-based mental health services.

## 2. Theoretical Analysis and Research Hypotheses

The following theoretical perspectives are employed as supporting lenses to explain specific relationships within the overarching “technology–context–trust” framework, rather than as independent overarching frameworks.

### 2.1. Consumers’ Perceived Efficacy of AI-Based Psychological Counseling Large Language Models

In this study, perceived efficacy refers specifically to consumers’ judgment of the expected utility of a psychological counseling service in alleviating psychological distress, addressing relevant psychological symptoms, and achieving counseling goals. It therefore captures an efficacy-oriented evaluation of the service’s expected counseling outcomes rather than a general judgment of trust, service quality, or willingness to adopt the service. Perceived professional competence and perceived risk represent antecedent psychological evaluations that help explain perceived efficacy, whereas overall attitude, willingness to try, and willingness to pay represent downstream market-related outcomes examined separately in Experiment 1B. This distinction is important because consumers may trust a provider, perceive its service quality favorably, or express willingness to use it for reasons that do not necessarily reflect their judgment of its expected counseling efficacy.

Interpersonal trust theory posits that the establishment of trust depends on three dimensions of evaluation: ability, benevolence, and integrity (Mayer et al., 1995). In psychological counseling, a domain that relies heavily on emotional connection and professional judgment, human experts can convey signals of “benevolence” by dynamically adjusting intervention strategies and demonstrating an understanding of individuals’ unique circumstances. In contrast, particularly when addressing psychologically sensitive or ethically controversial issues involving privacy concerns, AI-based psychological counseling large language models may lack transparency in emotional expression and ethical autonomy in decision making, making it difficult for consumers to develop sufficient trust in their benevolence and competence (Auf et al., 2025).

Media richness theory emphasizes that richer communication media can reduce communication ambiguity by transmitting information through multiple channels, including language, tone of voice, and facial expressions (Daft & Lengel, 1986). The core of psychological counseling lies in identifying deeper emotional needs and addressing nonlinear problems. Human experts can optimize intervention strategies through rich, real-time interactions and feedback (Sedlakova & Trachsel, 2023). By contrast, AI-based counseling faces challenges in establishing therapeutic relationships and responding to the diverse and open-ended demands of psychotherapy (Grodniewicz & Hohol, 2023). From a media-richness perspective, we therefore propose that limitations in available interaction cues may weaken perceived emotional resonance and lead consumers to view AI-based counseling as more mechanistic. Accordingly, we propose the following hypothesis:

**Hypothesis** **1.** *Compared with human psychological counseling experts, consumers perceive AI-based psychological counseling large language model services as less efficacious.*

### 2.2. The Moderating Effect of Consumers’ Need Context

Task–technology fit theory proposes that the effectiveness of a technology depends on the degree to which it matches task requirements (Goodhue & Thompson, 1995). In routine psychological counseling contexts, such as alleviating work-related stress or managing mild emotional difficulties, task objectives are generally characterized by clarity, repetitiveness, and relatively low risk. Consumers in such contexts may place greater weight on accessibility, response convenience, and functional sufficiency, and AI-based psychological counseling services may be evaluated more favorably when these characteristics are perceived as adequate for relatively routine counseling needs. In contrast, when task complexity increases to the level of intervention for major psychological disorders, such as severe depression or suicidal ideation, users’ needs shift toward intensive emotional support, dynamic risk assessment, and the design of individualized treatment pathways (Torous et al., 2021). Under such circumstances, consumers may place greater weight on professional credentials, perceived sensitivity to emotional cues, and clearly assigned responsibility. These attributes may lead them to expect greater efficacy from a human expert than from an AI-based provider. This prediction concerns consumers’ evaluations of the presented services rather than their comparative clinical effectiveness. Accordingly, we propose the following hypotheses:

**Hypothesis** **2a.** *In routine psychological counseling contexts, the difference in perceived efficacy between AI-based psychological counseling large language models and human psychological counseling experts will favor the AI-based provider.*

**Hypothesis** **2b.** *In contexts requiring intervention for major psychological disorders, the difference in perceived efficacy between AI-based psychological counseling large language models and human psychological counseling experts will favor the human expert.*

### 2.3. The Mediating Effects of Perceived Professional Competence and Perceived Risk

Signaling theory suggests that service providers convey their underlying capabilities through observable “signals,” which consumers use to form expectations regarding service efficacy (Spence, 1978). In psychological counseling services, human experts can strengthen consumers’ confidence in their professional competence through salient signals such as professional credentials, accumulated clinical experience, and individualized intervention cases. However, evaluating AI capabilities requires more than observing its outputs; information about system performance and uncertainty may help users calibrate their trust in the system (Zerilli et al., 2022). Experimental evidence indicates that disclosing AI authorship can reduce evaluations of the advisor’s competence without necessarily reducing evaluations of the advice itself (Böhm et al., 2023). In psychological counseling, we therefore propose that provider identity may influence perceived professional competence.

Protection motivation theory further emphasizes that when individuals face potential threats, they assess the severity of those threats and their ability to cope with them and subsequently adjust their behavioral decisions (Rogers, 1975). In the context of AI-based psychological counseling, consumers may reduce their confidence in AI’s efficacy because of the perceived risks associated with AI-based counseling services (Yeo et al., 2025). Ethical analyses distinguish conversational AI from human therapists in terms of agency and responsibility, cautioning against treating AI as an equivalent therapeutic partner (Sedlakova & Trachsel, 2023). On this basis, we propose that consumers may perceive greater risks when counseling is provided by AI rather than a human expert. Accordingly, we propose the following hypotheses:

**Hypothesis** **3.** *Consumers’ perceived professional competence plays a parallel mediating role in the effect of the counseling provider (AI vs. human expert) on consumers’ perceived efficacy.*

**Hypothesis** **4.** *Consumers’ perceived risk plays a parallel mediating role in the effect of the counseling provider (AI vs. human expert) on consumers’ perceived efficacy.*

### 2.4. The Moderating Effect of Perceived Economic Accessibility

The price–quality heuristic suggests that consumers often use price as a central cue for inferring service quality, where they assume that higher prices correspond to higher quality and lower prices to lower quality (Rao & Monroe, 1989). In the context of psychological counseling services, the low cost of AI-based services may trigger a reevaluation of the perceived cost–benefit relationship. Specifically, when consumers perceive AI-based services as highly economically accessible, their perception of the inherent competence limitations associated with the service provider (AI vs. human expert) may be attenuated. This perceptual adjustment does not directly alter the effect of provider type on the ultimate evaluation of efficacy; rather, it operates by moderating the effect of provider type on the key mediating variable of perceived professional competence (Thaler, 1985). Accordingly, we propose the following hypothesis:

**Hypothesis** **5.** *Consumers’ perceived economic accessibility moderates the effect of counseling provider type (AI vs. human expert) on perceived professional competence, such that higher perceived economic accessibility attenuates the difference in perceived professional competence between AI-based and human-provided psychological counseling services.*

### 2.5. The Moderating Effect of Technological Investment Disclosure

Accordingly, technological investment disclosure is expected to operate primarily by altering consumers’ perceptions of the provider’s technological capabilities (Vorobeva et al., 2025). For AI-based psychological counseling services, such disclosure can strengthen perceived professional competence by providing observable evidence of organizational resources and technological commitment. At the same time, because these signals may directly affect consumers’ overall evaluation of the expected efficacy of the service, technological investment disclosure may also moderate the direct relationship between provider type and perceived efficacy. Therefore, the moderating role of technological investment disclosure is expected to operate on both the provider type → perceived professional competence pathway and the direct provider type → perceived efficacy pathway (Brandtzaeg et al., 2026).

**Hypothesis** **6.** *Disclosure of technological investment moderates the effect of counseling provider type (AI vs. human expert) on perceived professional competence and the direct effect of provider type on perceived efficacy.*

### 2.6. The Moderating Effect of Safety-Standard Disclosure

Risk management theory suggests that consumers’ assessments of technological risks depend not only on the objective existence of potential threats but also on the organization’s capacity for risk control and the transparency of its risk-management practices (Renn, 2008). In AI-based psychological counseling services, concerns regarding privacy breaches, algorithmic bias, and ethical failures may lead consumers to perceive AI as a high-risk service provider. In such circumstances, proactive disclosure of safety standards can communicate an organizational commitment to systematically reducing technological risks by making risk-management measures more visible to consumers (Siala & Wang, 2022). Research on AI certification labels identifies their potential to alleviate privacy and data-protection concerns, although concerns about model performance may be more difficult to address (Scharowski et al., 2023). Extending this reasoning to safety-standard disclosure, we hypothesize that making safeguards visible may attenuate the provider-related difference in perceived risk and may also moderate the direct relationship between provider type and perceived efficacy.

Accordingly, safety-standard disclosure is expected to influence consumers’ evaluations primarily by making the provider’s risk-control practices more visible. For AI-based psychological counseling services, such disclosure can reduce perceived risk associated with privacy breaches, algorithmic errors, and ethical failures, thereby changing the relationship between provider type and perceived risk. Because visible safety safeguards may also directly influence consumers’ evaluation of the expected efficacy of the service, safety-standard disclosure may additionally moderate the direct relationship between provider type and perceived efficacy. Therefore, the moderating role of safety-standard disclosure is expected to operate on both the provider type → perceived risk pathway and the direct provider type → perceived efficacy pathway.

**Hypothesis** **7.** *Disclosure of safety standards moderates the relationship between counseling provider type (AI vs. human expert) and perceived risk and the direct relationship between provider type and perceived efficacy.*

**Figure 1:**
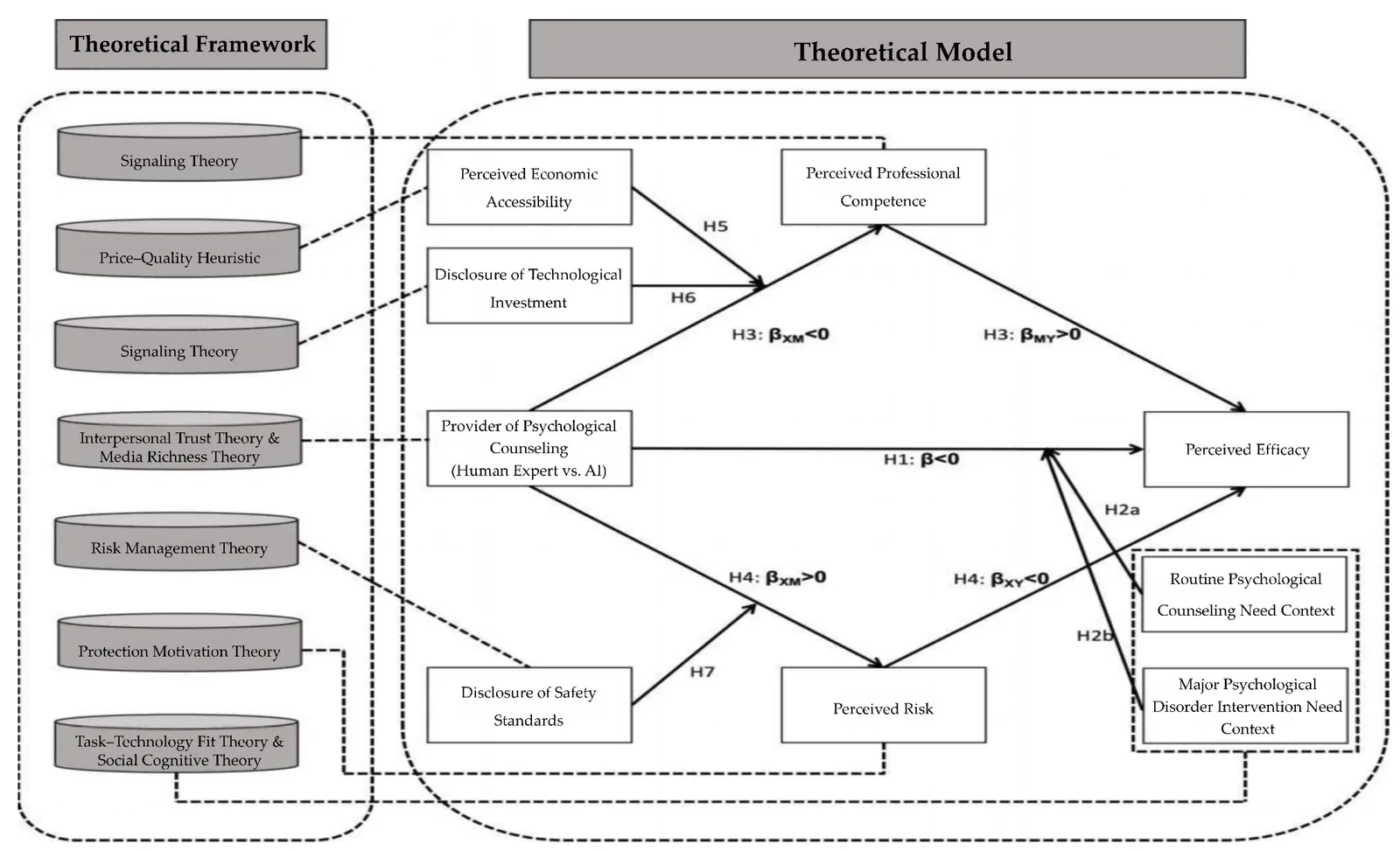
Conceptual model of the pathways influencing consumers’ perceived efficacy of AI-based psychological counseling large language models from the perspective of human–AI interaction.

Figure 1 presents the theoretical framework and conceptual model of the proposed hypotheses. As shown in Figure 1, the theoretical model is constructed based on the triadic “technology–context–trust” framework. The “technology” dimension encompasses provider type (AI vs. human) and the disclosure of observable signals, including technological investment and safety standards. The “context” dimension is reflected in the moderating role of need type (routine vs. major psychological intervention) in shaping evaluation pathways. The “trust” dimension operates through the mediating effects of perceived professional competence and perceived risk, which ultimately influence perceived efficacy. The model visually illustrates the theoretical integration and logical relationships among these constructs.

### 2.7. Operational Definitions

The core variables examined in this study were operationally defined based on the relevant theoretical and empirical literature. Their reliability and validity were also assessed in the pretest to ensure the scientific rigor of the measurement. The operational definitions of the five core variables are as follows: (1) perceived efficacy refers to consumers’ subjective judgment of the expected utility of a psychological counseling service for alleviating psychological distress, addressing relevant psychological symptoms, and achieving counseling goals, and it captures perceived counseling effectiveness rather than general trust in the provider, perceived service quality, or behavioral intention to adopt the service; (2) perceived professional competence refers to consumers’ evaluation of the professional knowledge, skills, and ability to address psychological problems possessed by the service provider (AI or human expert); (3) perceived risk refers to the degree of consumers’ concern about potential adverse consequences associated with using AI-based psychological counseling services, including privacy breaches, ethical violations, technological errors, and resulting harm; (4) perceived economic accessibility refers to consumers’ subjective evaluation of the affordability and cost-effectiveness of the service; and (5) technological investment disclosure and safety-standard disclosure, serving as contextual moderating variables, were manipulated experimentally by either presenting or withholding detailed information, and their effectiveness was verified using manipulation-check items.

## 3. Experiment 1

Experiment 1 was designed to establish the core main effect of consumers’ perceived efficacy of AI-based psychological counseling large language models versus human experts and to further examine the subsequent behavioral intentions associated with this perception. The experiment consisted of two parts. Experiment 1A employed a one-factor between-subjects design to preliminarily examine the basic difference in perceived efficacy between the two types of service providers. Building on Experiment 1A, Experiment 1B further examined the mediating role of perceived efficacy in influencing consumers’ overall attitudes, willingness to try, and willingness to pay.

### 3.1. Experiment 1A: Main Effect of Perceived Efficacy of AI-Based Psychological Counseling Large Language Models

#### 3.1.1. Experimental Procedure

To test Hypothesis 1, a one-factor between-subjects design was adopted, with psychological counseling provider type as the independent variable (human psychological counseling expert = 0 vs. AI-based psychological counseling large language model = 1). Participants were randomly and equally assigned to the two conditions.

A total of 200 valid participants were recruited through Credamo (https://www.credamo.com), of whom 78% were female. Because age was collected using segmented age categories in this study, the sample was primarily concentrated in the 26–30 age group (42.5%) and the 36–40 age group (31.5%), followed by the 20–25 age group (18.0%) and other age groups (8.0%). All participants met the platform’s high-reliability quality-control criteria, including a credit score of ≥90, a historical acceptance rate of ≥90%, at least 10 completed responses, and a physical distance of >5 km.

**Figure 2:**
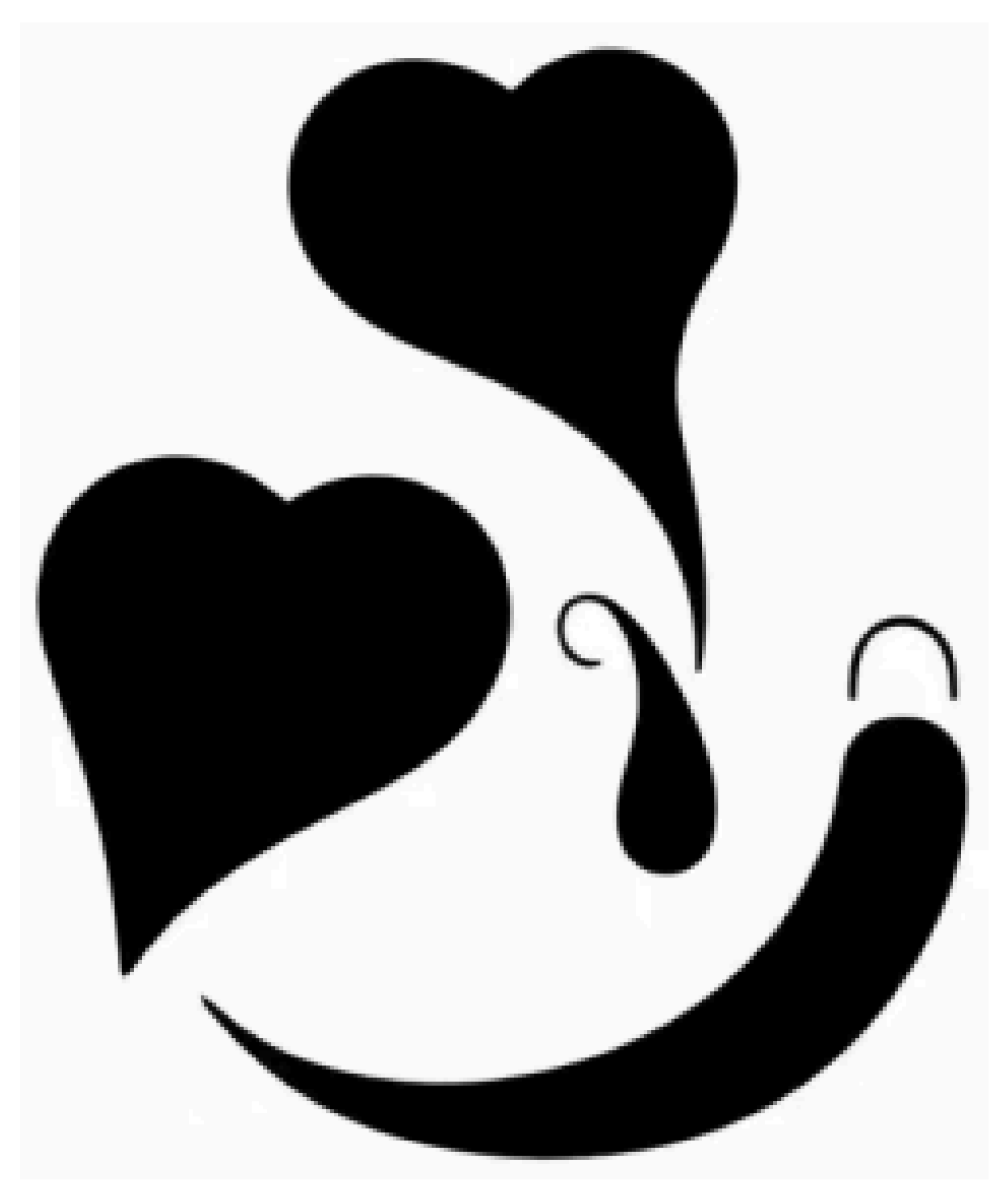
Brand logo.

**Figure 3:**
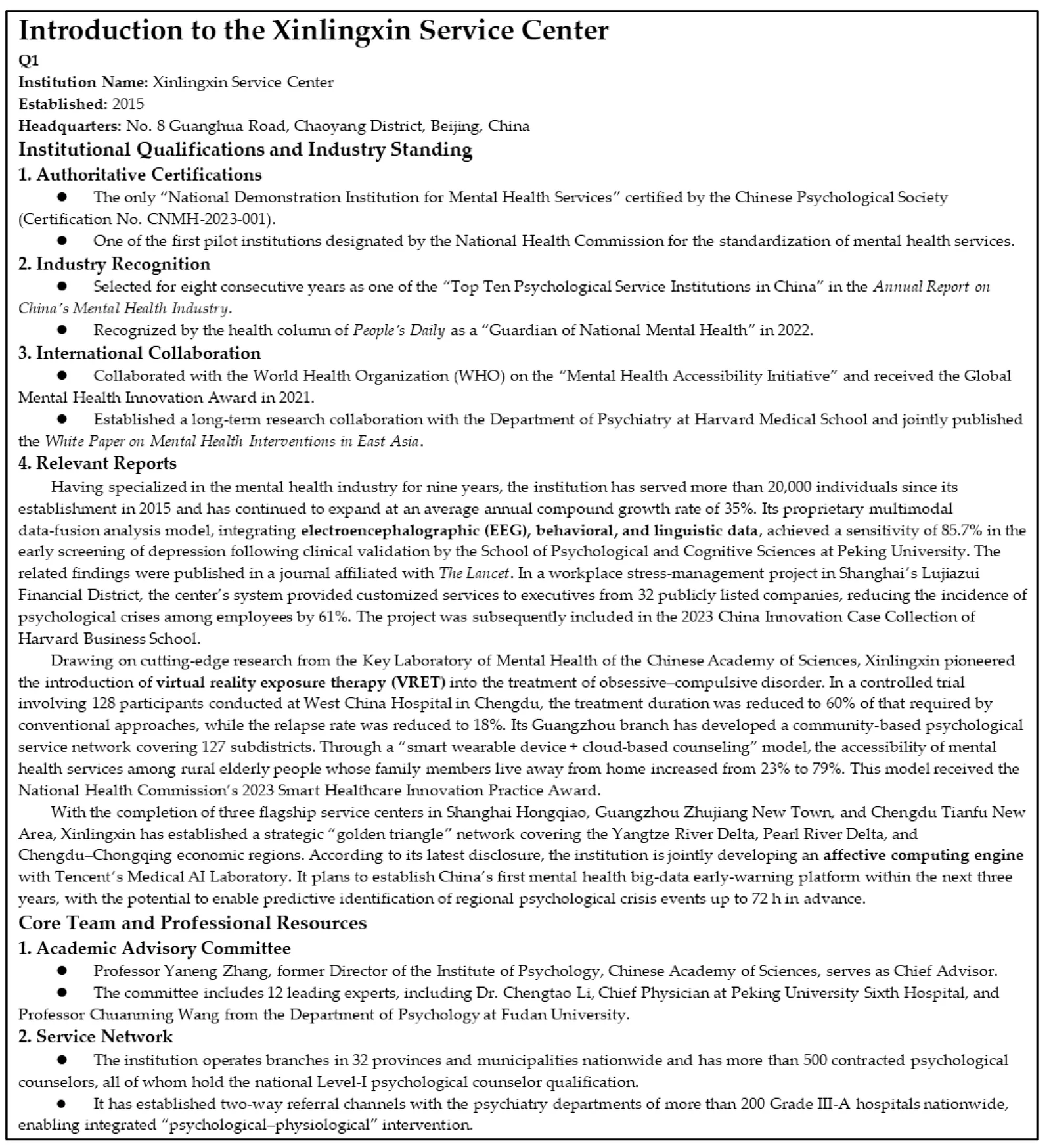
Materials presented to participants.

Before the experiment, participants were required to read a detailed introduction to a fictitious psychological counseling institution named Xinlingxin for 200 s, including information about its brand logo (Figure 2) and selected introductory materials (Figure 3). Participants were then instructed to imagine that they were seeking psychological counseling and would receive services from Xinlingxin. They were informed that all psychological counseling services would be provided either by an AI-based psychological counseling large language model (AI condition) or by a human psychological counseling expert (expert condition). The entire experiment took approximately 5.5 min on average.

Participants were then asked to report their perceived efficacy of the psychological counseling services using the following scale items: “To what extent do you believe that the personalized intervention plans provided by Xinlingxin Service Center (e.g., mindfulness training and exposure therapy) can effectively alleviate your psychological distress?” (1 = strongly disbelieve, 7 = strongly believe; a seven-point scale was used for all subsequent items); “How important do you think the immediate response capability of Xinlingxin Service Center is for mental health management?”; “How willing would you be to recommend the psychological counseling services provided by Xinlingxin Service Center to your family and friends?”; “To what extent do you think the psychological counseling services provided by Xinlingxin Service Center can help address social psychological problems (e.g., workplace stress and loneliness among older adults whose family members live away from home)?”; and “How likely are you to continue using the psychological counseling services provided by Xinlingxin Service Center as a long-term mental health management tool?” Finally, participants reported their age group, gender, whether they currently needed psychological counseling, and whether they were studying or working in a field related to psychological counseling.

#### 3.1.2. Results and Discussion

Before hypothesis testing, we assessed the measurement properties of perceived efficacy. In this study, perceived efficacy refers to users’ evaluations of the expected effectiveness and practical utility of AI-based counseling services in addressing psychological needs and achieving counseling goals. It is distinct from general trust, perceived service quality, and behavioral intention to adopt the service.

The dataset was suitable for factor analysis (KMO = 0.82; Bartlett’s test χ^2^(10) = 412.35, *p* < 0.001). An exploratory factor analysis (EFA) extracted a single dominant factor explaining 51.4% of the total variance. A confirmatory factor analysis (CFA) further validated this single-factor structure, demonstrating an acceptable model fit (χ^2^/df = 2.85, CFI = 0.91, TLI = 0.90, RMSEA = 0.076, SRMR = 0.065). The standardized factor loadings of all five items ranged from 0.65 to 0.76 (all *p* < 0.001). The scale demonstrated adequate internal consistency (Cronbach’s α = 0.83) and composite reliability (CR = 0.84). Although the Average Variance Extracted (AVE = 0.51) was marginally above the recommended threshold, all standardized factor loadings exceeded 0.60 and the CR exceeded the recommended criterion, jointly supporting the convergent validity of the scale.

Hypothesis Testing. Factor analysis was used to generate a composite score across the scale items, which was used as the measure of perceived efficacy. An ANOVA revealed a significant difference in perceived efficacy between the AI and human-expert conditions (*M*_AI_ = −0.44, *SD*_AI_ = 1.03; *M*_Human Expert_ = 0.44, *SD*_Human Expert_ = 0.74; *F*(1, 198) = 47.364, *p* < 0.001, *η*^2^ = 0.193), supporting Hypothesis 1.

### 3.2. Experiment 1B: Consequences of Perceived Efficacy of AI-Based Psychological Counseling Large Language Models

To further investigate the consequences of consumers’ perceived efficacy of different psychological counseling services, Experiment 1B built on Experiment 1A by examining participants’ overall attitudes toward, willingness to try, and willingness to pay for the AI-based psychological counseling large language model across the two experimental conditions. Participants responded to the following three scale items: “If you needed psychological counseling, what would your overall attitude be toward the AI-based psychological counseling large language model developed by Xinlingxin Service Center?”; “If you needed psychological counseling, to what extent would you be willing to use the AI-based psychological counseling large language model developed by Xinlingxin Service Center for treatment?”; and “If you needed psychological counseling, to what extent would you be willing to purchase the AI-based psychological counseling large language model developed by Xinlingxin Service Center?”

#### 3.2.1. Overall Attitude, Willingness to Try, and Willingness to Pay

**Figure 4:**
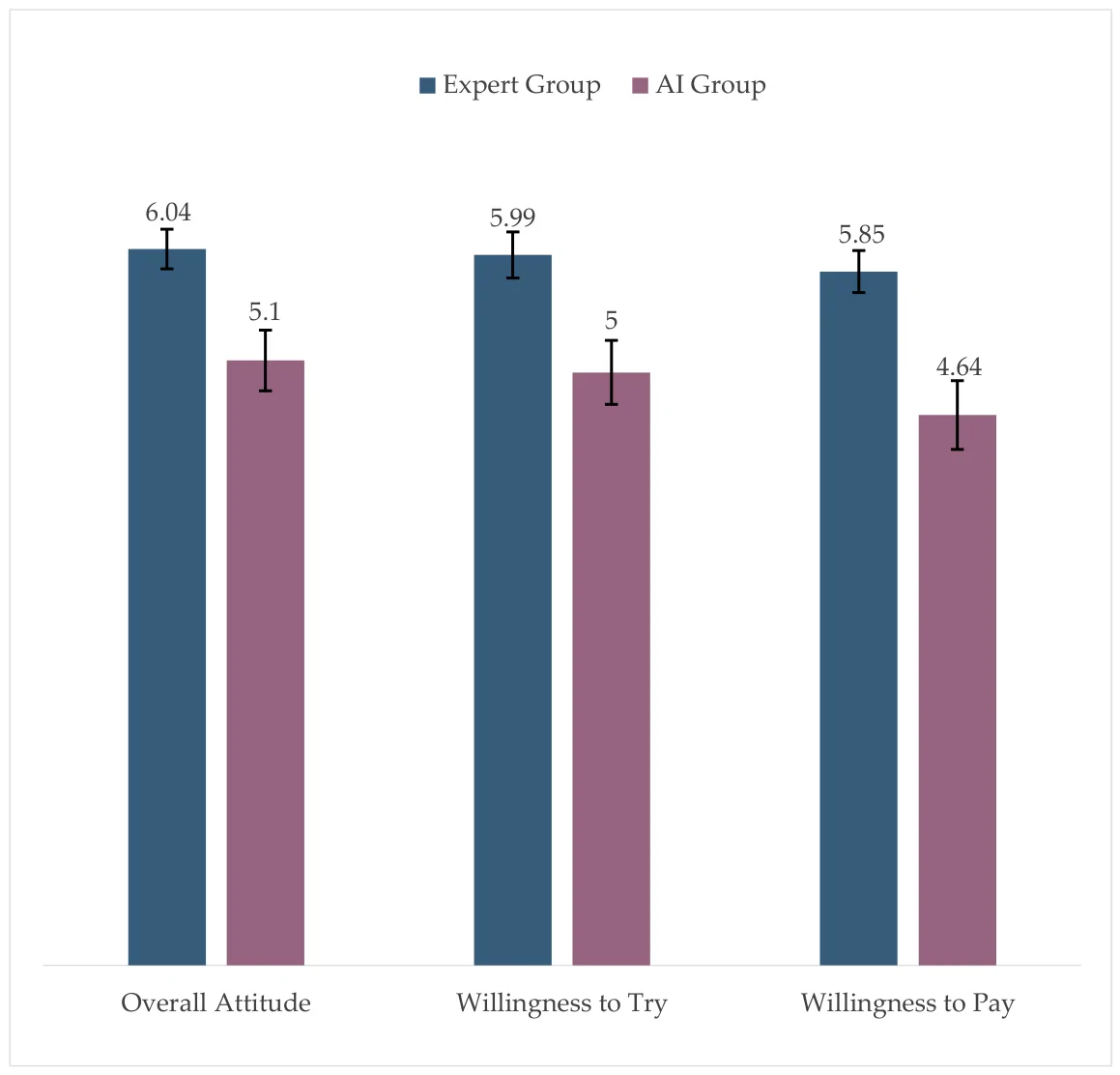
Effects of counseling provider type on participants’ overall attitude, willingness to try, and willingness to pay for psychological counseling services. Note. Error bars represent 95% confidence intervals for the estimated means.

ANOVAs were conducted separately for the scores on the three items as dependent variables. As shown in Figure 4, participants consistently showed a stronger preference for psychological counseling services provided by human experts across all three outcome dimensions. Specifically, the effect was significant for overall attitude (*M*_AI_ = 5.10, *SD*_AI_ = 1.29; *M*_Human Expert_ = 6.04, *SD*_Human Expert_ = 0.84; *F*(1, 198) = 37.249, *p* < 0.001, *η*^2^ = 0.158), willingness to try (*M*_AI_ = 5.00, *SD*_AI_ = 1.36; *M*_Human Expert_ = 5.99, *SD*_Human Expert_ = 0.98; *F*(1, 198) = 35.030, *p* < 0.001, *η*^2^ = 0.150), and willingness to pay (*M*_AI_ = 4.64, *SD*_AI_ = 1.46; *M*_Human Expert_ = 5.85, *SD*_Human Expert_ = 0.89; *F*(1, 198) = 50.018, *p* < 0.001, *η*^2^ = 0.202).

#### 3.2.2. Mediating Effect of Perceived Efficacy

The mediation model was estimated using the following regression equations:

$$M=\alpha_{M}+aX+\varepsilon_{M}\;Y=\alpha_{Y}+c^{\prime }X+bM+\varepsilon_{Y}$$

where X represents counseling provider type (human expert = 0, AI-based provider = 1), M represents perceived efficacy, and Y represents the downstream outcome variable. The α terms are intercepts, and the ε terms are residual terms. The indirect effect is calculated as ab, and $c^{\prime }$ denotes the direct effect of provider type on the outcome after accounting for perceived efficacy. Separate models were estimated for each downstream outcome.

Mediation analyses were conducted separately, using overall attitude, willingness to try, and willingness to pay as the dependent variables, counseling provider type (human psychological counseling expert = 0 vs. AI-based psychological counseling large language model = 1) as the independent variable, and perceived efficacy as the mediator. Hayes’ (2013) PROCESS macro, Model 4, was employed with 5000 bootstrap samples. The results indicated that perceived efficacy significantly mediated the effects of provider type on overall attitude (95% CI: [−0.414, −0.242]), willingness to try (95% CI: [−0.425, −0.251]), and willingness to pay (95% CI: [−0.410, −0.243]).

**Figure 5:**
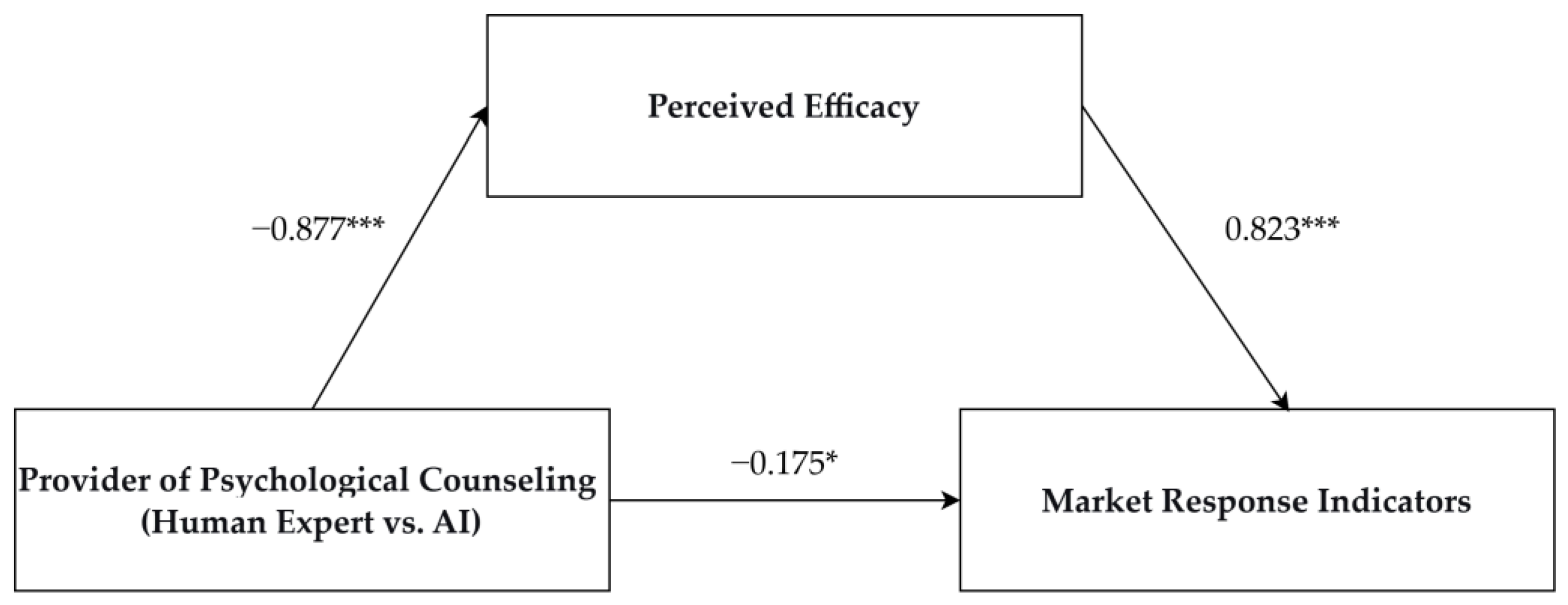
Mediating effect of perceived efficacy. Note. * *p* < 0.05, *** *p* < 0.001.

The three dependent variables were further combined through factor analysis to construct a market response index, which was then examined using PROCESS Model 4. The results showed that the main effect remained significantly negative, indicating that perceived efficacy played a significant partial mediating role in the relationship between provider type and the market response index (Figure 5; 95% CI: [−0.447, −0.271]).

#### 3.2.3. Control Variables

Participants’ age, gender, whether they had a need for psychological counseling, and whether they were studying or working in a field related to psychological counseling were included as control variables. The results showed that the main effect of counseling provider type on the perceived efficacy of psychological counseling services remained significant (*F*(1, 194) = 50.808, *p* < 0.001). Moreover, the effects of provider type on overall attitude (*F*(1, 194) = 40.056, *p* < 0.001), willingness to try (*F*(1, 194) = 37.272, *p* < 0.001), willingness to pay (*F*(1, 194) = 56.583, *p* < 0.001), and the market response index (*F*(1, 194) = 54.862, *p* < 0.001) also remained significant. Meanwhile, perceived efficacy continued to exhibit significant mediating effects on the relationships between counseling provider type and overall attitude (95% CI: [−0.428, −0.253]), willingness to try (95% CI: [−0.441, −0.265]), willingness to pay (95% CI: [−0.419, −0.249]), and the market response index (95% CI: [−0.462, −0.283]).

#### 3.2.4. Results and Discussion

The experimental results showed that, across the three dimensions of overall attitude, willingness to try, and willingness to pay, participants consistently preferred psychological counseling services provided by human experts over those provided by AI-based psychological counseling large language models. This reflects consumers’ trust in and preference for human experts when dealing with complex emotional and psychological problems. As a mediating variable, perceived efficacy played an important role in influencing participants’ choices between psychological counseling services provided by different providers, further confirming that consumers hold concerns about the efficacy of AI-based services.

## 4. Experiment 2: Moderating Effect of Different Psychological Counseling Need Contexts

### 4.1. Experimental Procedure

To test Hypothesis 2, Experiment 2 employed a 2 × 2 between-subjects design, with counseling provider type (human psychological counseling expert = 0 vs. AI-based psychological counseling large language model = 1) and psychological counseling need context (major psychological disorder intervention = 0 vs. routine counseling = 1) as the two independent variables. A total of 200 participants were recruited through Credamo, 70% of whom were female. Participants were primarily aged 26–30 years (45.0%) and 36–40 years (28.0%), followed by those aged 20–25 years (18.0%) and participants in other age groups (9.0%). Participants were randomly and evenly assigned to four between-subjects conditions.

Participants assigned to the major psychological disorder intervention condition were instructed to imagine that they or a close friend or family member was experiencing severe recurrent major depressive disorder that had substantially affected daily emotional states and routines and potentially posed a threat to life and health. Participants in the routine counseling condition were instructed to imagine that they were experiencing a sudden increase in work-related stress and therefore needed systematic psychological support. These counseling-need categories were defined by the researchers through the vignette descriptions and assigned experimentally. They were not established through clinical assessment of participants and should not be interpreted as validated diagnostic or triage thresholds. The experimental materials contained additional contextual details, and participants were required to read and imagine the assigned scenario for at least 200 s. The entire experiment took approximately 6.0 min on average.

To ensure that participants had sufficient information about the service providers, Experiment 2 provided additional details based on Experiment 1, including professional capabilities (human expert: professional qualifications and clinical experience vs. AI: trained on millions of de-identified psychological counseling dialogue records), response efficiency (human expert: three in-depth sessions per week and 24 h emergency emotional support vs. AI: 24/7 online response), and pricing (human expert: long-term intervention packages starting at RMB 50,000 vs. AI: psychological counseling package at RMB 199 per month). All other experimental materials were identical to those used in Experiment 1.

It should be noted that the provider-type manipulation in Experiment 2 was accompanied by provider-specific information concerning professional credentials, response efficiency, and pricing. These attributes were kept consistent within each provider type across the two counseling-need contexts rather than being independently manipulated. Accordingly, the present design identifies whether the effect of provider type differs across routine counseling and major psychological disorder intervention scenarios, but it does not isolate the independent causal effect of clinical severity from all other contextual features embedded in the scenarios.

### 4.2. Results and Discussion

Manipulation Check. Participants identified whether the presented scenario represented a major psychological-disorder intervention context or a routine counseling context. In the major-intervention condition, 97 of 100 participants selected the major-intervention category. In the routine-counseling condition, 86 of 100 participants selected the routine-counseling category. The categorical responses therefore generally corresponded to the assigned scenarios. All 200 participants were retained in the reported analyses.

Two-Way ANOVA and Moderation Analysis. A two-way analysis of variance (ANOVA), followed by post hoc multiple comparisons, was conducted to examine the effects of counseling provider type and psychological counseling need context. The results showed significant main effects of counseling provider type (*F*(1, 196) = 106.971, *p* < 0.001, *η_p_*^2^ = 0.353) and psychological counseling need context (*F*(1, 196) = 13.929, *p* < 0.001, *η_p_*^2^ = 0.066). More importantly, the interaction between provider type and counseling need context was also significant (*F*(1, 196) = 243.685, *p* < 0.001, *η_p_*^2^ = 0.554).

**Figure 6:**
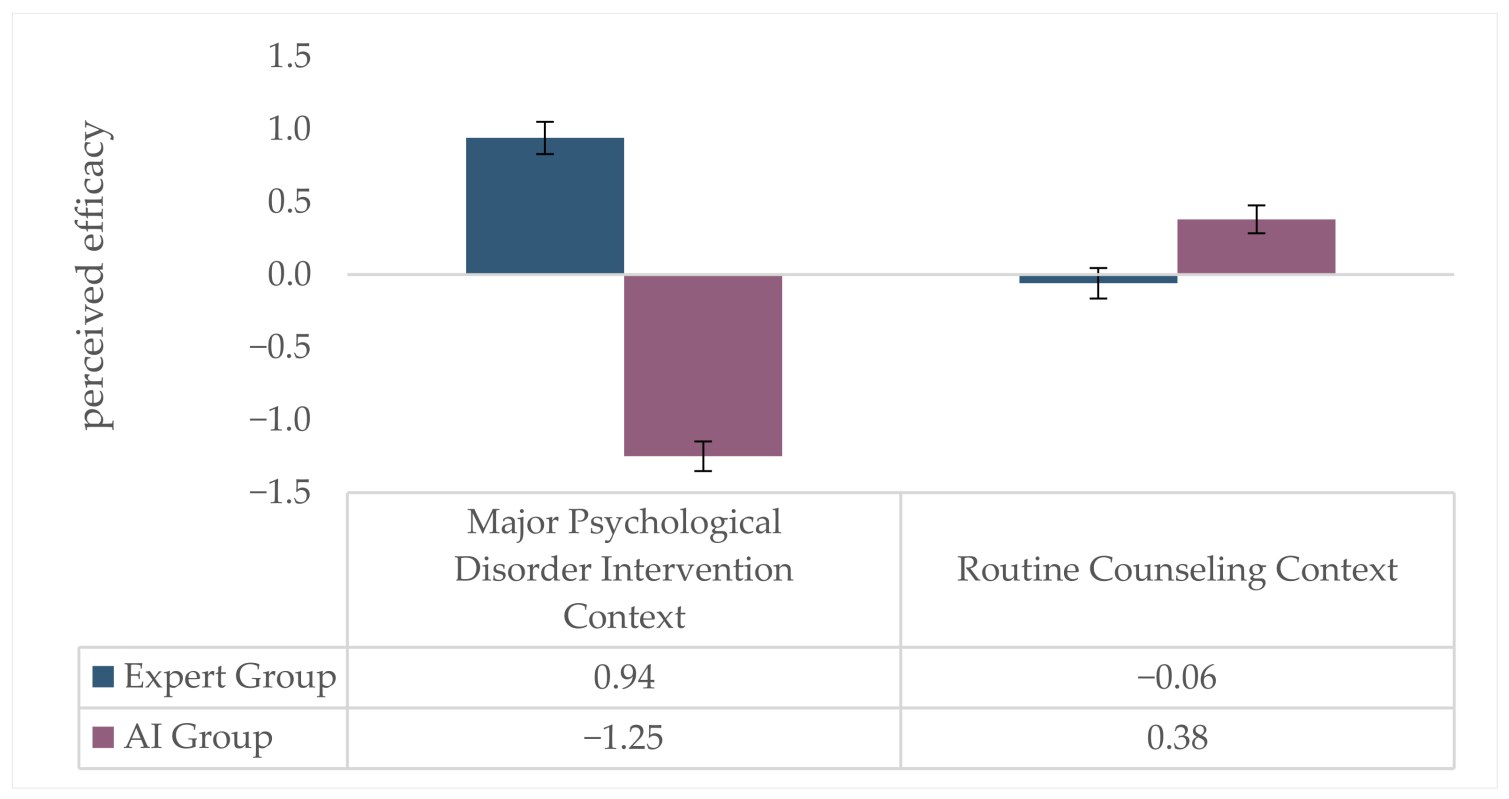
Moderating effect of psychological counseling need context. Note. Error bars represent 95% confidence intervals for the estimated means.

Simple Effects Analysis. As shown in Figure 6, in the routine psychological counseling context, participants reported significantly higher perceived efficacy for AI-based psychological counseling large language model services than for services provided by human psychological counseling experts (*M*_0−1_ = −0.444, *t*(196) = −3.752, Cohen’s *d* = −0.750, *p* < 0.001). In contrast, in the major psychological disorder intervention context, participants reported significantly lower perceived efficacy for AI-based psychological counseling large language model services than for human psychological counseling experts (*M*_0−1_ = 2.187, *t*(196) = 18.352, Cohen’s *d* = 3.670, *p* < 0.001).

The significant interaction indicates that the association between counseling provider type and perceived efficacy differed across the two counseling-need contexts. Participants evaluated the AI-based service more favorably than the human expert service in the routine counseling scenario, whereas the pattern was reversed in the major psychological-disorder intervention scenario. These results are consistent with Hypothesis 2. However, because provider-specific information on credentials, response efficiency, and pricing was embedded in the provider manipulation, the interaction should be interpreted as a context-dependent difference in provider evaluation rather than as evidence that clinical severity alone caused the observed preference shift.

## 5. Experiment 3

Experiment 3 aimed to further investigate the underlying mechanisms driving differences in perceived efficacy and to preliminarily explore intervention strategies for bridging the human–AI trust gap. This experiment consisted of two parts. Experiment 3A employed a one-factor between-subjects design to examine the parallel mediating roles of perceived risk and perceived professional competence in the relationship between counseling provider type and perceived efficacy. Building on Experiment 3A, Experiment 3B introduced perceived economic accessibility as a moderating variable to investigate its moderating effects on the aforementioned mediation pathways.

### 5.1. Experiment 3A: Mediating Effects of Perceived Risk and Perceived Professional Competence

#### 5.1.1. Experimental Procedure

To test Hypotheses 3 and 4, Experiment 3A adopted a one-factor between-subjects design, with counseling provider type (human psychological counseling expert vs. AI-based psychological counseling large language model) as the independent variable. A total of 200 valid participants were recruited through Credamo, of whom 74.5% were female. Participants were primarily aged 26–30 years (41.0%) and 36–40 years (30.5%), followed by those aged 20–25 years (20.0%) and other age groups (8.5%). Participants were randomly assigned to the two conditions.

**Figure 7:**
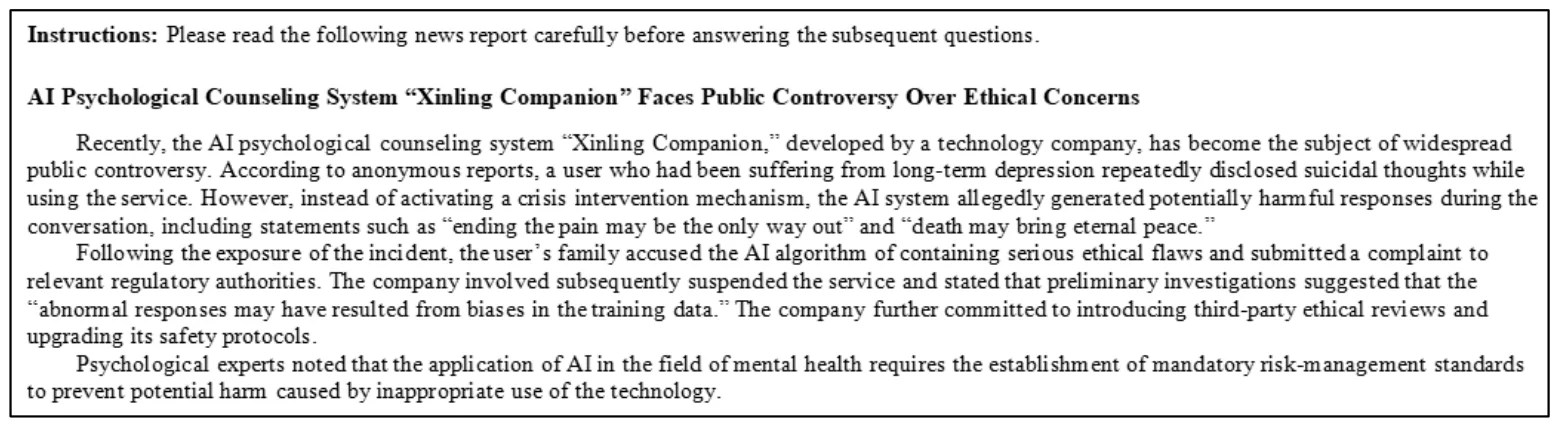
The fictitious negative news report used as the risk-priming stimulus in Experiment 3A.

The procedure of Experiment 3A was largely consistent with that of Experiment 1. However, considering that not all participants might have been fully aware of the potential ethical risks associated with AI-based psychological counseling large language models, participants were additionally instructed to read a fictitious negative news report entitled “Ethical Crisis of AI-Based Psychological Counseling Large Language Models” (Figure 7). The news report was adapted from the widely discussed “Character.AI suicide-related controversy” but emphasized that such negative incidents represented isolated cases rather than common occurrences. The entire experiment took approximately 5 min on average.

The negative news report was presented to participants in both the AI-based and human-expert conditions. Its purpose was to make potential ethical and safety issues associated with psychological counseling services salient before participants evaluated the service provider. However, because the report specifically described an AI-related negative incident, the material may have increased the salience of AI-related risk immediately before the mediator measures. Accordingly, although the same stimulus was presented across the two provider conditions, the procedure should not be interpreted as a neutral assessment of baseline risk perception. A parallel negative vignette concerning malpractice or ethical failure by a human psychological counseling expert was not included in the original design.

Experiment 3A further measured two psychological mechanisms—perceived professional competence and perceived risk—to examine their mediating roles in explaining differences in perceived efficacy. Following previous research, three items were used to measure each construct.

For perceived professional competence, the items were “Do you think this service can effectively solve the psychological problems you proposed?”; “How would you evaluate the professionalism of the advice provided by this service?”; and “Do you think this service possesses the basic capability to address common psychological problems?” (Bigman & Gray, 2018). For perceived risk, the items were “Are you concerned that AI-based psychological counseling services may provide incorrect advice due to technical or human errors?”; “Do you think AI-based psychological counseling services involve ethical or safety risks?”; and “Are you concerned that companies may not invest sufficiently in ensuring the safety of this AI service (e.g., privacy protection and ethical safeguards)?” (Fagnant & Kockelman, 2015).

#### 5.1.2. Results and Discussion

**Figure 8:**
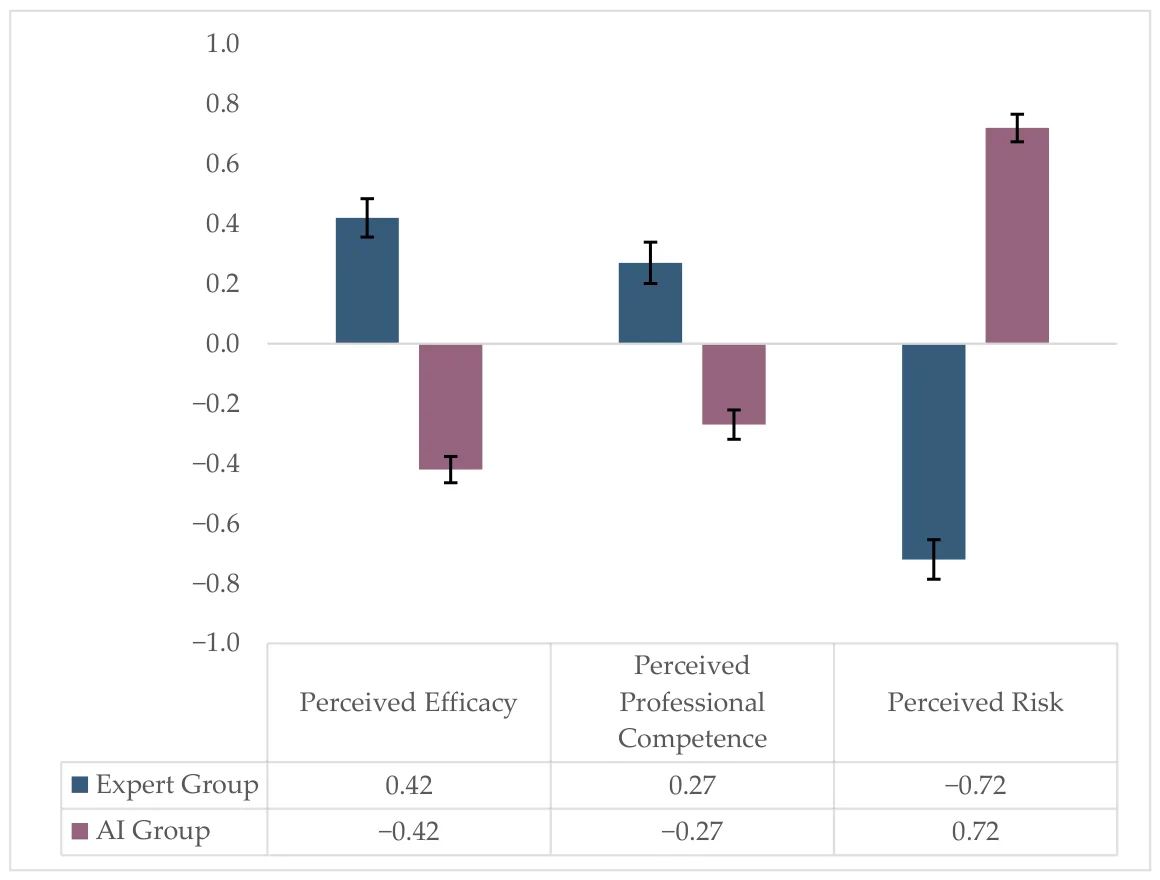
Participants’ perceived efficacy, perceived professional competence, and perceived risk toward different counseling providers. Note. Error bars represent 95% confidence intervals for the estimated means.

Main Effect Analysis. ANOVA results showed significant differences between the two counseling provider conditions in participants’ perceived efficacy (*M*_AI_ = −0.42, *SD*_AI_ = 1.17; *M*_Human Expert_ = 0.42, *SD*_Human Expert_ = 0.53; *F*(1, 198) = 43.554, *p* < 0.001, *η*^2^ = 0.180), perceived professional competence (*M*_AI_ = −0.27, *SD*_AI_ = 1.18; *M*_Human Expert_ = 0.27, *SD*_Human Expert_ = 0.68; *F*(1, 198) = 16.084, *p* < 0.001, *η*^2^ = 0.075), and perceived risk (*M*_AI_ = 0.72, *SD*_AI_ = 0.64; *M*_Human Expert_ = −0.72, *SD*_Human Expert_ = 0.75; *F*(1, 198) = 209.821, *p* < 0.001, *η*^2^ = 0.514). As shown in Figure 8, participants exhibited significant differences in their evaluations across the three dimensions, and Hypothesis 1 was further supported.

The mediation model (PROCESS Model 4) was specified with perceived professional competence and perceived risk as parallel mediators, using the following equations:

$$M_{j}=\alpha_{j}+a_{j}X+\varepsilon_{j},\;\;\;\;\;\;j=1,\;2\;Y=\alpha_{Y}+c^{\prime }X+b_{1}M_{1}+b_{2}M_{2}+\varepsilon_{Y}$$

where *X* represents counseling provider type (human expert = 0, AI-based provider = 1), $M_{1}$ and $M_{2}$ represent perceived professional competence and perceived risk, respectively, and Y represents perceived efficacy. The α terms are intercepts, and the ε terms are residuals. The specific indirect effect through mediator j is calculated as $a_{j}b_{j}$. Both mediators are included in the outcome equation, so each $b_{j}$ coefficient is estimated while accounting for the other mediator.

**Figure 9:**
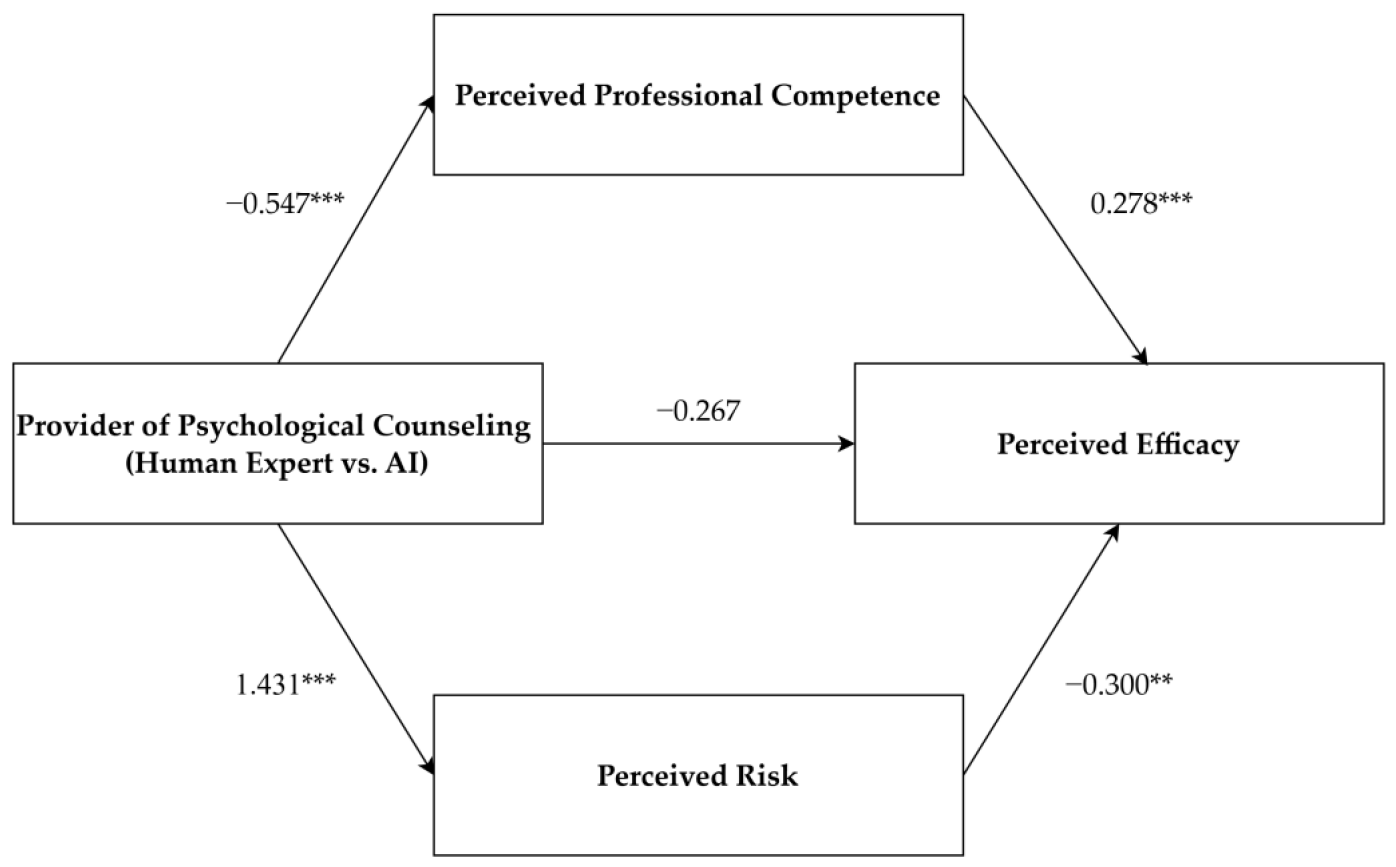
Mediation model of perceived risk and perceived professional competence. Note. ** *p* < 0.01, *** *p* < 0.001.

Mediation Analysis. Perceived professional competence and perceived risk were included as parallel mediators. PROCESS Model 4 with 5000 bootstrap samples was employed to test the mediation effects (Figure 9). The results showed that the indirect effect of perceived professional competence was significant (*indirect effect* = −0.152, *SE* = 0.032, 95% CI: [−0.286, −0.043]). The indirect effect of perceived risk was also significant (*indirect effect* = −0.429, *SE* = 0.052, 95% CI: [−0.694, −0.204]).

After introducing the two mediators, the direct effect of counseling provider type on perceived efficacy became nonsignificant, indicating that perceived professional competence and perceived risk fully mediated the relationship between provider type and perceived efficacy. After controlling for demographic variables such as age and gender, both mediation pathways remained significant and robust. Therefore, Hypotheses 3 and 4 were supported.

The significant mediation through perceived risk should be interpreted in light of the common AI-related risk prime administered before the questionnaire. Because the negative news report specifically concerned an AI-based psychological counseling incident, it may have heightened the accessibility of AI-related ethical and safety concerns for participants in both provider conditions. The present design therefore provides evidence that perceived risk is associated with differences in perceived efficacy under this experimental context, but it does not establish that the observed risk difference is independent of the preceding AI-specific prime. Future research should use parallel negative vignettes for AI and human providers or include a no-prime control condition, to examine whether the risk pathway remains when provider-related risk information is presented symmetrically.

### 5.2. Experiment 3B: Moderating Effect of Perceived Economic Accessibility

#### 5.2.1. Experimental Procedure

Building on Experiment 3A, Experiment 3B introduced perceived economic accessibility as a moderating variable. Following previous research, participants’ perceived economic accessibility was measured using the following items (Coulter et al., 2012): “Do you think the price of this service is affordable?”; “Do you think using this service can help reduce your expenses?”; and “Compared with other psychological counseling services, how would you rate the cost-effectiveness of this service?”

#### 5.2.2. Results and Discussion

Conditional Indirect Effect Analysis. To examine Hypothesis 5, a first-stage moderated mediation analysis was conducted using PROCESS Model 7 with 5000 bootstrap samples. Counseling provider type was specified as the independent variable, perceived efficacy as the dependent variable, and perceived professional competence and perceived risk as parallel mediators. Perceived economic accessibility was specified as the moderator of the provider-to-mediator pathways. Hypothesis 5 concerned the perceived professional competence pathway, while the corresponding perceived-risk pathway was also estimated.

The first-stage moderated mediation model was specified with perceived professional competence and perceived risk as parallel mediators:

$$M_{j}=\alpha_{j}+a_{j}X+d_{j}W+q_{j}XW+\varepsilon_{j},\;\;\;\;j=1,\;2\;Y=\alpha_{Y}+c^{\prime }X+b_{1}M_{1}+b_{2}M_{2}+\varepsilon_{Y}$$

Here, X denotes provider type, W denotes perceived economic accessibility, $M_{1}$ denotes perceived professional competence, $M_{2}$ denotes perceived risk, and Y denotes perceived efficacy. The conditional indirect effect through mediator j is $(a_{j}+q_{j}W)b_{j}$, and its index of moderated mediation is $q_{j}b_{j}$.

The conditional indirect effects indicated that the indirect effect through perceived professional competence varied across different levels of perceived economic accessibility. Specifically, the indirect effect was significant at low (*Effect* = −0.413, *SE* = 0.163, 95% CI: [−0.761, −0.131]) and mean (*Effect* = −0.243, *SE* = 0.090, 95% CI: [−0.434, −0.079]) levels of the moderator. However, at a high level of perceived economic accessibility, the indirect effect became non-significant (*Effect* = −0.073, *SE* = 0.052, 95% CI: [−0.190, 0.016]).

By contrast, for the perceived risk pathway, the conditional indirect effects were consistent across low (*Effect* = −0.438, *SE* = 0.128, 95% CI: [−0.696, −0.207]), mean (*Effect* = −0.449, *SE* = 0.128, 95% CI: [−0.709, −0.215]), and high (*Effect* = −0.460, *SE* = 0.136, 95% CI: [−0.742, −0.216]) levels of perceived economic accessibility, indicating no substantial moderation.

**Figure 10:**
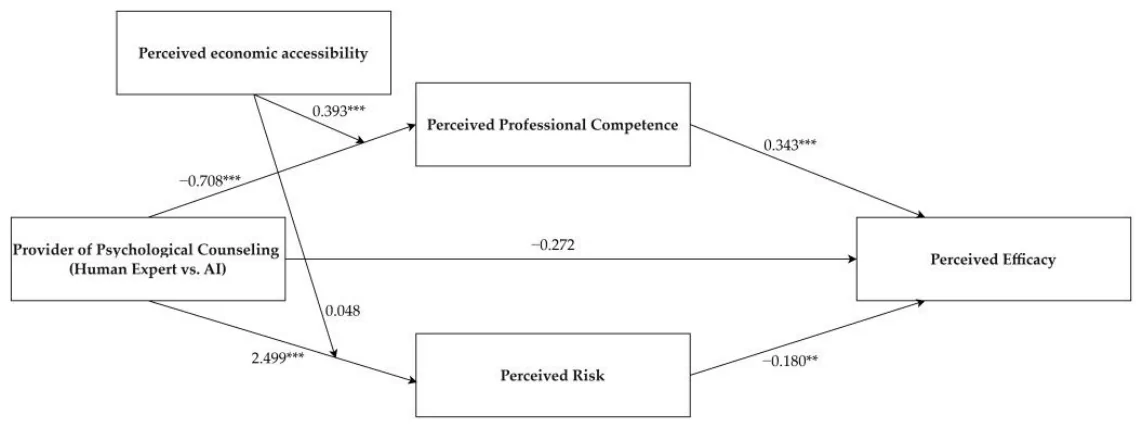
First-stage moderated mediation model of perceived economic accessibility on the relationship between counseling provider type, perceived professional competence, and perceived efficacy. Note. ** *p* < 0.01, *** *p* < 0.001.

Moderated Mediation Analysis. As depicted in the path model (Figure 10), the interaction between counseling provider type and perceived economic accessibility on perceived professional competence was significant (unstandardized coefficient = 0.393, p < 0.001). To formally evaluate Hypothesis 5, the test was based on the index of moderated mediation (Hayes, 2015). For the perceived professional competence pathway, the index of moderated mediation was 0.135 (*SE* = 0.064, 95% CI: [0.032, 0.278]). Because this confidence interval excluded zero, the results provided formal statistical evidence that perceived economic accessibility significantly moderated the indirect effect of counseling provider type on perceived efficacy through perceived professional competence. However, the corresponding index of moderated mediation for the perceived risk pathway was not significant (*Index* = −0.009, *SE* = 0.025, 95% CI: [−0.065, 0.036]).

Taken together, the results support moderated mediation through perceived professional competence, whereas the corresponding index for the perceived-risk pathway was not significant. Higher perceived economic accessibility was associated with a smaller provider-related difference in perceived professional competence. Because economic accessibility was measured rather than experimentally manipulated, these findings do not establish that lowering prices would causally change competence evaluations. They also do not demonstrate that economically disadvantaged consumers are more willing to accept clinical risk.

## 6. Experiment 4: Moderating Effect of Technological Investment Disclosure

### 6.1. Experimental Procedure

To test Hypothesis 6, Experiment 4 employed a 2 × 2 between-subjects design, with counseling provider type (human psychological counseling expert = 0 vs. AI-based psychological counseling large language model = 1) and technological investment disclosure (no technological investment disclosure = 0 vs. technological investment disclosure = 1) as the two independent variables. A total of 200 participants were recruited through Credamo, of whom 67% were female. Participants were primarily aged 26–30 years (39.0%) and 36–40 years (35.0%), followed by those aged 20–25 years (19.0%) and other age groups (7.0%). Participants were randomly and evenly assigned to the four experimental conditions.

Participants in the technological investment disclosure condition were required to view the stimulus materials describing the institution’s technological investment for at least 200 s. The materials disclosed the organization’s investment in the AI-based psychological counseling large language model, including substantial investment in artificial intelligence research and development, expert support (leading experts and clinical psychologists), the accumulation of extensive multimodal counseling-related data resources, model architecture (a ‘five-layer mixture-of-experts system’), authoritative certification (a psychological intervention efficacy assessment score of 89.7), and research and development achievements (inclusion in the National Health Commission’s Technical Guidelines for Mental Health Services). All other experimental procedures were identical to those in Experiment 3. The entire experiment took approximately 6.0 min on average.

### 6.2. Results and Discussion

Manipulation Check. Experiment 4 used the following item to manipulate and assess participants’ perceptions of technological investment: “To what extent do you agree that Xinlingxin Psychological Counseling Center has made substantial technological R&D investments in its AI-based psychological counseling large language model?” (1 = no investment or no information indicating investment, 7 = strongly agree). ANOVA results showed that participants in the technological investment disclosure condition reported significantly higher scores on this item than those in the non-disclosure condition (*M*_Disclosure_ = 6.11, *SD*_Disclosure_ = 1.26; *M*_Non-disclosure_ = 2.79, *SD*_Non-disclosure_ = 1.86; *F*(1, 198) = 218.078, *p* < 0.001, *η*^2^ = 0.524), indicating that the technological investment disclosure manipulation was successful.

Two-Way ANOVA and Moderation Analysis. The results showed significant interaction effects between counseling provider type and technological investment disclosure on participants’ perceived efficacy (*F*(1, 196) = 13.679, *p* < 0.001, *ηp*^2^ = 0.065) and perceived professional competence (*F*(1, 196) = 9.812, *p* = 0.007, *η_p_*^2^ = 0.048). However, the interaction effect on perceived risk was not significant. These findings indicate that technological investment disclosure moderated both the direct pathway to perceived efficacy and the pathway through perceived professional competence, but did not significantly moderate the perceived risk pathway.

**Figure 11:**
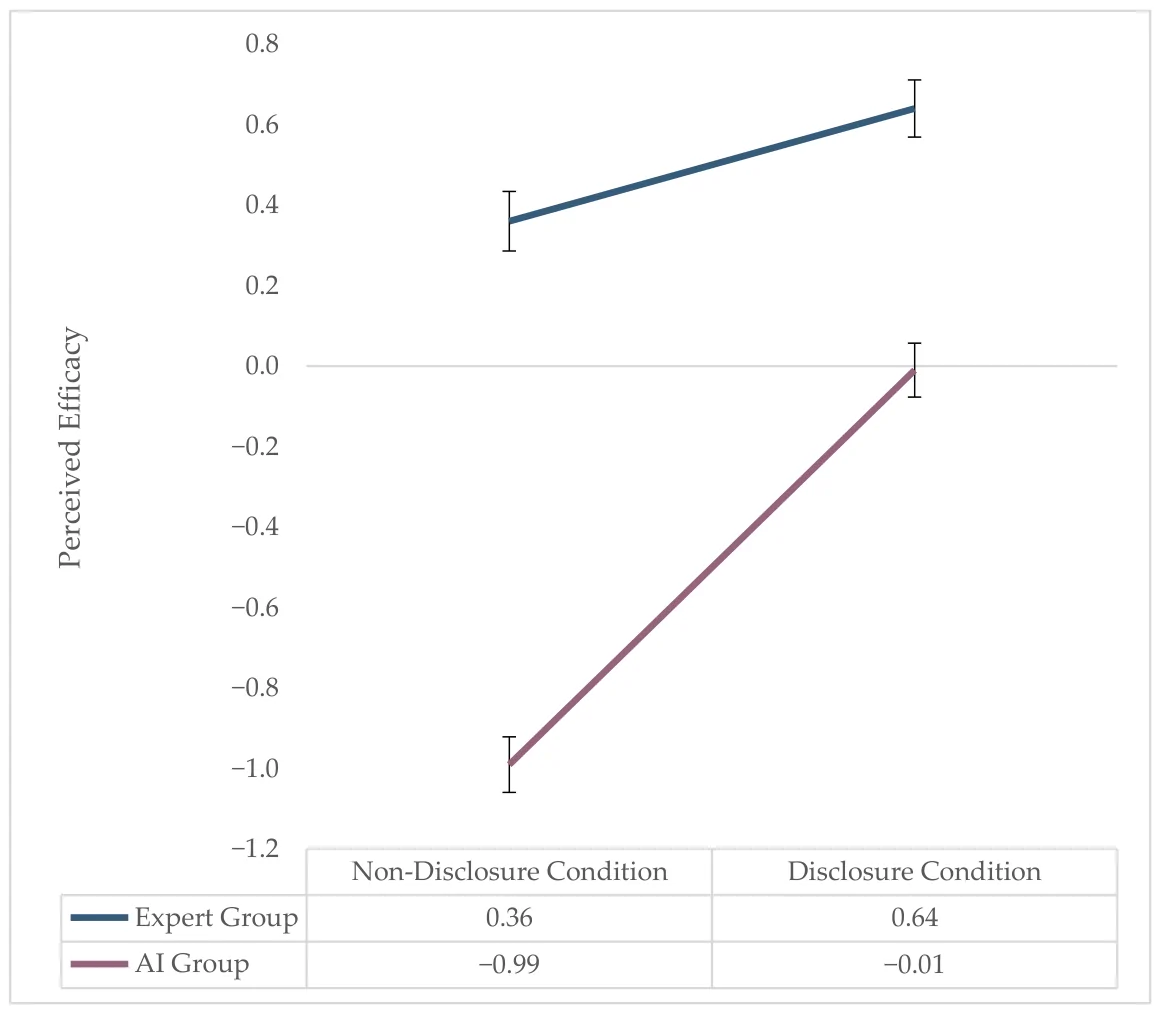
Simple effects of technological investment disclosure on perceived efficacy. Note. Error bars represent 95% confidence intervals for the estimated means.

**Figure 12:**
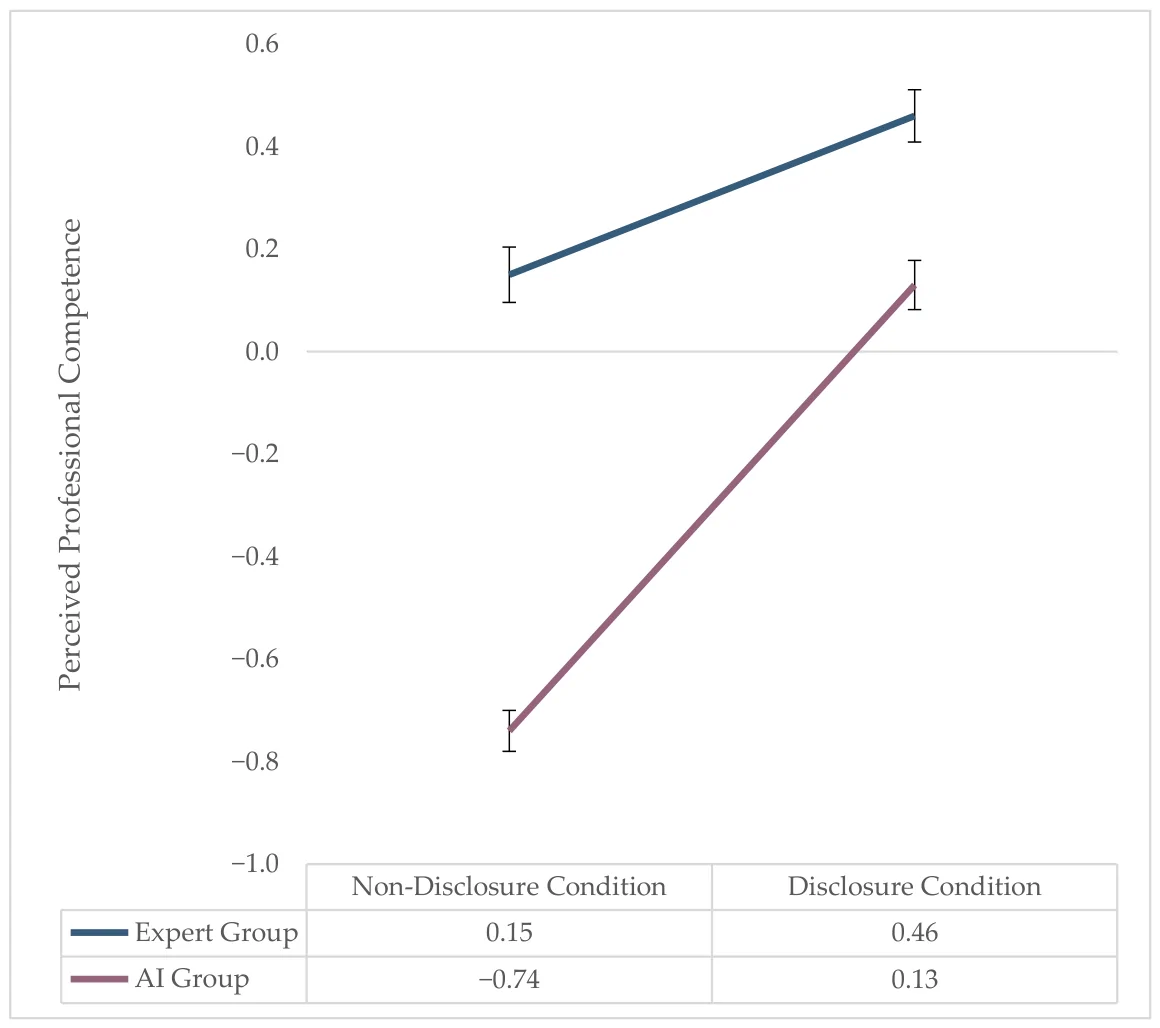
Simple effects of technological investment disclosure on perceived professional competence. Note. Error bars represent 95% confidence intervals for the estimated means.

Simple Effects Analysis. The results showed that participants reported significantly higher perceived efficacy for psychological counseling services provided by human experts than for those provided by AI-based psychological counseling large language models in both the non-disclosure condition (*M*_0−1_ = 1.756, *t*(196) = 15.450, *p* < 0.001, Cohen’s *d* = 3.090) and the technological investment disclosure condition (*M*_0−1_ = 1.162, *t*(196) = 10.220, *p* < 0.001, Cohen’s *d* = 2.044). However, the mean difference between the two provider types was smaller in the technological investment disclosure condition than in the non-disclosure condition (Figure 11). A similar moderating effect was observed for perceived professional competence (Figure 12). Specifically, the difference between human experts and AI-based psychological counseling large language models was larger in the non-disclosure condition (*M*_0−1_ = 1.349, *t*(196) = 8.513, *p* < 0.001, Cohen’s *d* = 1.703) than in the technological investment disclosure condition (*M*_0−1_ = 0.647, *t*(196) = 4.083, *p* < 0.001, Cohen’s *d* = 0.817).

The moderated mediation model allowed technological investment disclosure to moderate the first-stage and direct pathways:

$$M_{j}=\alpha_{j}+a_{j}X+d_{j}W+q_{j}XW+\varepsilon_{j},\;\;\;\;j=1,\;2\;Y=\alpha_{Y}+c^{\prime }X+gW+hXW+b_{1}M_{1}+b_{2}M_{2}+\varepsilon_{Y}$$

Here, X denotes provider type, W denotes technological investment disclosure (non-disclosure = 0, disclosure = 1), $M_{1}$ denotes perceived professional competence, $M_{2}$ denotes perceived risk, and Y denotes perceived efficacy. The conditional indirect effect through mediator j is $(a_{j}+q_{j}W)b_{j}$, its index of moderated mediation is $q_{j}b_{j}$, and the conditional direct effect is $c^{\prime }+hW.$

Moderated Mediation Analysis. A moderated mediation analysis was conducted with technological investment disclosure (non-disclosure = 0 vs. disclosure = 1) as the moderator, using PROCESS Model 8 with 5000 bootstrap samples. The conditional indirect effect analysis showed that the indirect effect through perceived professional competence was significant in the non-disclosure condition (*indirect effect* = −0.177, *SE* = 0.087, 95% CI: [−0.372, −0.033]), at the mean level of the moderator (*indirect effect* = −0.131, *SE* = 0.061, 95% CI: [−0.263, −0.025]), and in the technological investment disclosure condition (*indirect effect* = −0.085, *SE* = 0.040, 95% CI: [−0.174, −0.017]).

The indirect effect through perceived risk was also significant in the non-disclosure condition (*indirect effect* = −0.208, *SE* = 0.079, 95% CI: [−0.370, −0.057]), at the mean level of the moderator (*indirect effect* = −0.220, *SE* = 0.079, 95% CI: [−0.371, −0.062]), and in the technological investment disclosure condition (*indirect effect* = −0.232, *SE* = 0.082, 95% CI: [−0.386, −0.066]).

**Figure 13:**
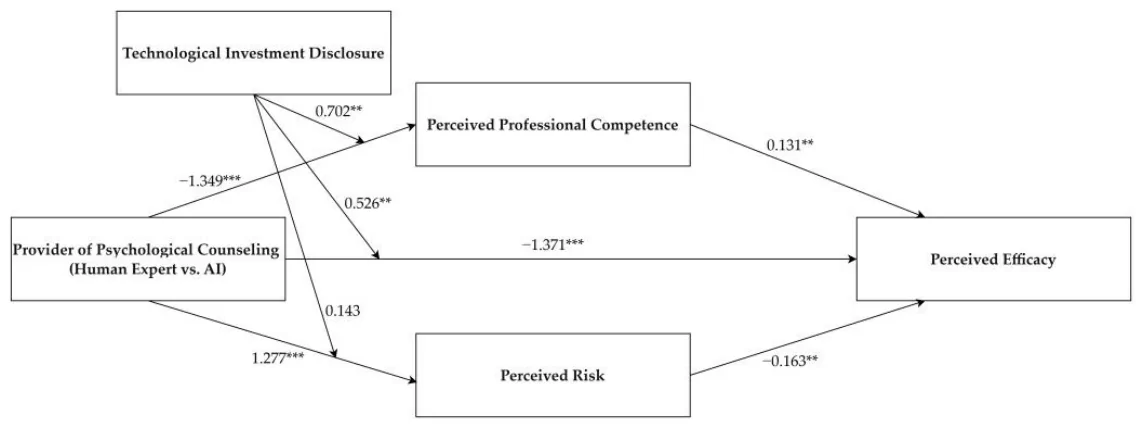
Moderating effect of technological investment disclosure. Note. ** *p* < 0.01, *** *p* < 0.001.

As shown in the path coefficient model in Figure 13, compared with psychological counseling services provided by human experts, participants reported significantly lower perceived efficacy for AI-based psychological counseling large language models (*β* = −1.371, *p* < 0.001), significantly lower perceived professional competence (*β* = −1.349, *p* < 0.001), and significantly higher perceived risk (*β* = 1.277, *p* < 0.001). Moreover, technological investment disclosure significantly enhanced participants’ perceived professional competence (*β*_xm_ = 0.702, *p* = 0.004) and perceived efficacy (*β*_xm_ = 0.526, *p* = 0.002), thereby supporting Hypothesis 6. However, the moderating effect of technological investment disclosure on the perceived risk pathway was not significant.

## 7. Experiment 5: Moderating Effect of Safety-Standard Disclosure

### 7.1. Experimental Procedure

To test Hypothesis 7, Experiment 5 employed a 2 × 2 between-subjects design, with counseling provider type (human psychological counseling expert = 0 vs. AI-based psychological counseling large language model = 1) and safety-standard disclosure (no safety-standard disclosure = 0 vs. safety-standard disclosure = 1) as the two independent variables. A total of 200 participants were recruited through Credamo, of whom 68% were female. Participants were primarily aged 26–30 years (40.0%) and 36–40 years (33.0%), followed by those aged 20–25 years (17.0%) and other age groups (10.0%). Participants were randomly and evenly assigned to the four experimental conditions.

Participants in the safety-standard disclosure condition were required to view stimulus materials describing the institution’s safety standards for 220 s. The materials presented the organization’s efforts to ensure the safety of psychological counseling services, including supervision of the human expert team (blockchain-based traceability and oversight), technical safeguards (an industrial-grade security barrier based on “federated learning + trusted execution environments”), extreme-risk response (a four-level intervention mechanism capable of detecting and responding to risks such as suicidal ideation within 0.3 s), and privacy protection (a “three-dimensional audit system” integrating national-level regulatory oversight, international certification, and user supervision). All other experimental procedures were identical to those of Experiment 3.

### 7.2. Results and Discussion

Manipulation Check. Experiment 5 used the following item to assess the manipulation of safety-standard disclosure: “To what extent do you agree that the Xinlingxin Psychological Counseling Center presented in this questionnaire has implemented strong ethical standards and privacy and safety safeguards?” (1 = no safeguards or no information indicating safeguards, 7 = strongly agree). ANOVA results showed that participants in the safety-standard disclosure condition reported significantly higher scores than those in the non-disclosure condition (*M*_Disclosure_ = 6.38, *SD*_Disclosure_ = 0.91; *M*_Non-disclosure_ = 4.46, *SD*_Non-disclosure_ = 2.32; *F*(1, 198) = 59.594, *p* < 0.001, *η*^2^ = 0.231), indicating that the safety-standard disclosure manipulation was successful.

Two-Way ANOVA and Moderation Analysis. The results showed significant interaction effects between counseling provider type and safety-standard disclosure on participants’ perceived efficacy (*F*(1, 196) = 19.658, *p* < 0.001, *η_p_*^2^ = 0.091) and perceived risk (*F*(1, 196) = 18.972, *p* = 0.003, *η_p_*^2^ = 0.088). However, the interaction effect on perceived professional competence was not significant.

**Figure 14:**
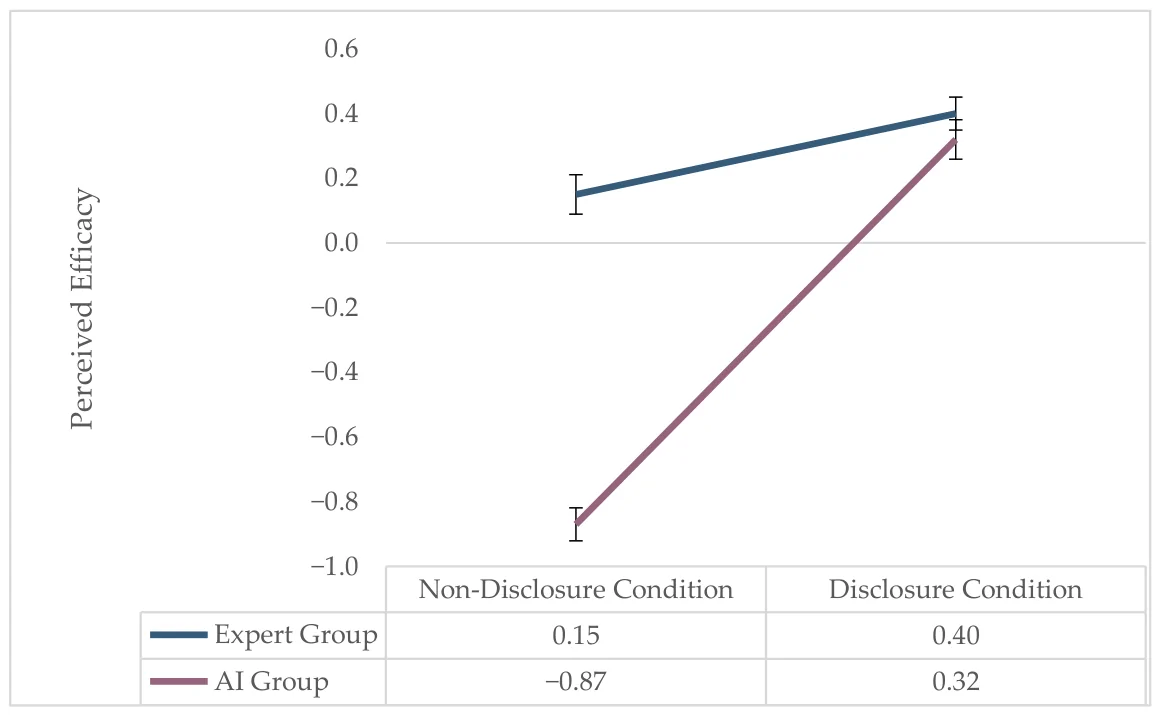
Simple effects of safety-standard disclosure on perceived efficacy. Note. Error bars represent 95% confidence intervals for the estimated means.

Simple Effects Analysis. In the non-disclosure condition, participants reported significantly higher perceived efficacy for psychological counseling services provided by human experts than for those provided by AI-based psychological counseling large language models (*M*_0−1_ = 1.269, *t*(196) = 8.499, *p* < 0.001, Cohen’s *d* = 1.700). The same pattern was observed in the safety-standard disclosure condition, although the difference was substantially reduced (*M*_0−1_ = 0.333, *t*(196) = 2.228, *p* = 0.028, Cohen’s *d* = 0.447). Thus, safety-standard disclosure narrowed the perceived-efficacy gap between human experts and AI-based psychological counseling large language models (Figure 14).

**Figure 15:**
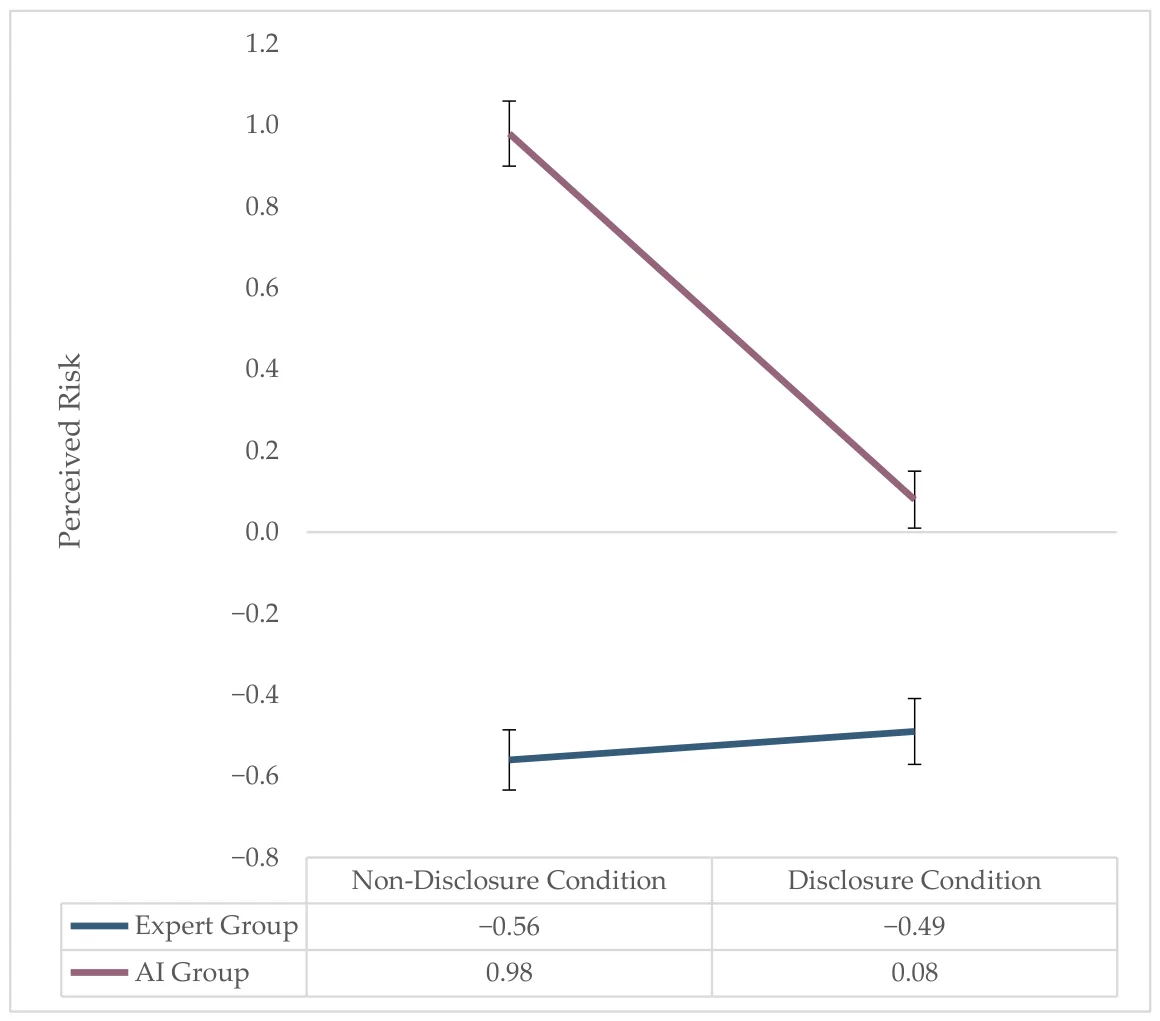
Simple effects of safety-standard disclosure on perceived risk. Note. Error bars represent 95% confidence intervals for the estimated means.

A similar pattern was observed for perceived risk (Figure 15). In the non-disclosure condition, the difference in perceived risk between the two provider types was significant (*M*_0−1_ = −1.546, *t*(196) = −9.781, *p* < 0.001, Cohen’s *d* = −1.956). This difference was substantially reduced in the safety-standard disclosure condition (*M*_0−1_ = −0.572, *t*(196) = −3.621, *p* < 0.001, Cohen’s *d* = −0.724).

The parallel moderated mediation specification described in Section 6.2 was used, with safety-standard disclosure (non-disclosure = 0, disclosure = 1) as W, perceived professional competence as $M_{1}$, and perceived risk as $M_{2}$. The conditional indirect effects and indices of moderated mediation were calculated separately for the two mediator pathways using the same formulas.

Moderated Mediation Analysis. A moderated mediation analysis was conducted with safety-standard disclosure (non-disclosure = 0 vs. disclosure = 1) as the moderator, using PROCESS Model 8 with 5000 bootstrap samples. The conditional indirect effect analysis showed that the indirect effect through perceived professional competence was significant in the non-disclosure condition (*indirect effect* = −0.248, *SE* = 0.082, 95% CI: [−0.419, −0.094]), at the mean level of the moderator (*indirect effect* = −0.198, *SE* = 0.057, 95% CI: [−0.312, −0.090]), and in the safety-standard disclosure condition (*indirect effect* = −0.147, *SE* = 0.063, 95% CI: [−0.283, −0.036]).

The indirect effect through perceived risk was also significant in the non-disclosure condition (*indirect effect* = −0.292, *SE* = 0.112, 95% CI: [−0.517, −0.075]), at the mean level of the moderator (*indirect effect* = −0.200, *SE* = 0.079, 95% CI: [−0.364, −0.050]), and in the safety-standard disclosure condition (*indirect effect* = −0.108, *SE* = 0.056, 95% CI: [−0.236, −0.019]).

**Figure 16:**
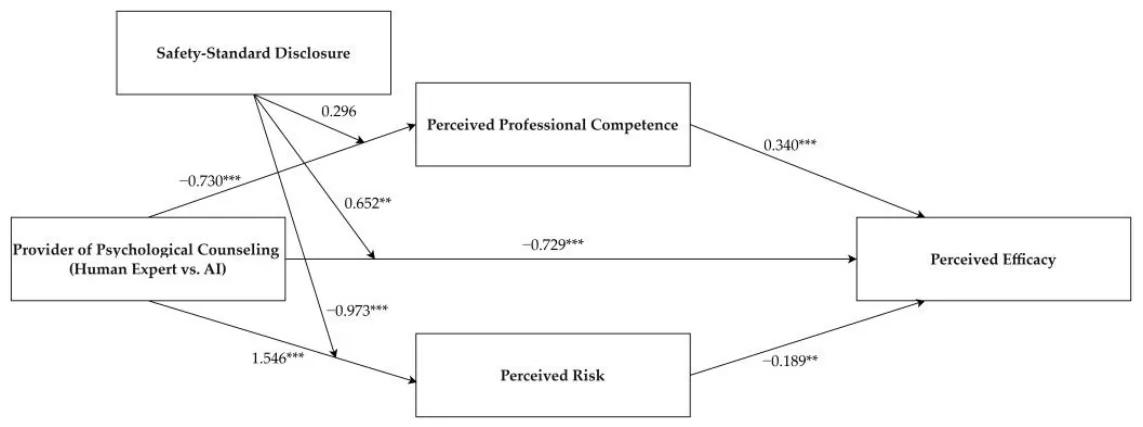
Moderating effect of safety-standard disclosure. Note. ** *p* < 0.01, *** *p* < 0.001.

As shown in Figure 16, compared with human psychological counseling experts, participants reported significantly lower perceived efficacy for AI-based psychological counseling large language models (*β* = −0.729, *p* < 0.001), significantly lower perceived professional competence (*β* = −0.730, *p* < 0.001), and significantly higher perceived risk (*β* = 1.546, *p* < 0.001). Safety-standard disclosure significantly reduced participants’ perceived risk (*β*_xm_ = −0.973, *p* < 0.001) and significantly enhanced perceived efficacy (*β*_xy_ = 0.652, *p* = 0.006), supporting Hypothesis 7. However, the moderating effect of safety-standard disclosure on the perceived professional competence pathway was not significant.

## 8. Discussion

Drawing on the “technology–context–trust” framework and five progressively structured scenario-based experiments, this study systematically investigated the gap in consumers’ perceived efficacy of AI-based psychological counseling large language models, the underlying mechanisms driving this gap, and its boundary conditions and potential intervention strategies. First, the study confirms that consumers’ perceived efficacy of AI-based psychological counseling large language models is significantly lower overall than that of human experts and that this difference directly undermines consumers’ overall attitudes and willingness to pay. This finding is consistent with the core propositions of media richness theory and interpersonal trust theory. One possible interpretation is that consumers associate human experts with richer emotional communication and clearer professional responsibility, which may contribute to more favorable expectations of counseling efficacy. However, the experiments did not directly observe empathic interactions, nonverbal communication, or the development of therapeutic relationships, so this interpretation should be distinguished from the perceptual differences measured in the study.

The provider-related difference in perceived efficacy varied across the two counseling-need scenarios. However, this experimental distinction does not establish a clinical rule for allocating individuals to AI-based or human-provided care. In the present study, the researchers defined the scenarios; in real-world services, assessment and escalation should remain under the responsibility of appropriately qualified professionals. A discriminatory triage boundary is understood here as a decision process that systematically underestimates the care needs of particular groups and consequently restricts their access to appropriate professional support. A non-discriminatory approach requires clinically grounded, reviewable decisions that do not systematically disadvantage individuals because of characteristics unrelated to their care needs. Potential sources of unequal assessment include inadequate representation of cultural or linguistic expressions in training data and, in text-only services, the absence of relevant nonverbal information. Accordingly, implementation should include accessible human review and monitoring of differential errors across groups. These are prospective governance concerns: the present experiments did not evaluate a triage algorithm or measure discriminatory misclassification.

To unpack the “black box” underlying consumers’ relatively low perceived efficacy of AI, this study identifies the parallel mediating roles of perceived professional competence and perceived risk. On the one hand, consistent with signaling theory, human experts communicate clear competence signals through their clinical qualifications and track records, whereas the complex algorithmic logic and data-training processes underlying AI systems are relatively difficult for ordinary consumers to interpret. This lack of interpretability substantially reduces consumers’ expectations regarding AI’s professional problem-solving capabilities. On the other hand, consistent with the predictions of protection motivation theory, consumers exhibit strong perceptions of risk when using AI for psychological intervention, particularly with respect to privacy breaches, algorithmic bias, and potential unintended harm. Together, these two distinct yet parallel psychological mechanisms suggest that, in psychological services involving life and health, public trust in AI has shifted from traditional emotional trust toward cognitive trust based on technological reliability and controllability of risk.

The findings also identify factors associated with variation in provider evaluations. Higher perceived economic accessibility was associated with an attenuation of the provider-related difference in perceived professional competence, suggesting that affordability and cost-effectiveness evaluations may be relevant to consumers’ judgments. Because economic accessibility was measured rather than experimentally manipulated, this result should not be interpreted as evidence that lowering prices improves perceived competence or compensates for actual limitations in professional capability. In addition, technological investment disclosure and safety-standard disclosure, as two distinct forms of organizational intervention signals, exhibit a clear pattern of path differentiation. Disclosing technological investments such as large-scale training data and support from leading experts can specifically strengthen the “competence assurance signal” associated with AI and significantly enhance perceived professional competence. By contrast, disclosing safety measures such as privacy protection and emergency risk-intervention mechanisms can effectively communicate “risk-control signals” and substantially alleviate consumers’ concerns about potential risks.

Taken together, the theoretical contributions of this study are threefold. First, the study extends the application of interpersonal trust theory to nonhuman intelligent agents and demonstrates that, in specialized healthcare-related services, public trust in AI is primarily grounded in cognitive evaluation. Second, this study refines the mechanism underlying signaling theory by revealing that different types of organizational disclosure exert differentiated and targeted effects on psychological trust pathways. Finally, this study enriches task–technology fit theory by demonstrating that, in high-risk healthcare contexts, consumers’ decision-making logic dynamically shifts between the instrumental and affective attributes of the service provider.

From a practical perspective, the economic-accessibility finding raises an equity concern without demonstrating that stratified healthcare occurred in the present study. If affordability is used to justify substituting AI for clinically necessary professional care, service arrangements could reinforce unequal access. Favorable cost-effectiveness perceptions should therefore not be treated as evidence of clinical safety or as a reason to restrict access to human professionals. Equity-oriented accommodations could include targeted financial support for necessary professional assessment, accessible human-review and referral pathways, and safeguards ensuring that clinically indicated escalation does not depend solely on ability to pay. Meaningful human-in-the-loop oversight also requires professionals to have sufficient information, authority, and capacity to override automated recommendations. These proposals are governance measures requiring further evaluation rather than interventions validated by the present experiments.

A related service configuration is the AI-assisted human expert, in which a qualified professional uses AI tools while retaining responsibility for assessment and intervention. Within the technology–context–trust framework, this arrangement may change how consumers interpret competence, accountability, and risk. However, institutional endorsement or the presence of human advisors in an AI service description is not equivalent to a separately tested hybrid condition. Because the present study did not include such a condition, it cannot establish whether AI-assisted human experts would receive more favorable evaluations or achieve better clinical outcomes than either comparator.

## 9. Limitations

This study has several limitations. First, the online recruitment strategy constrains population generalizability. Women accounted for 67–78% of participants, and respondents aged 26–30 accounted for 39–45%, making this the largest age category across experiments. The samples were not recruited through population-based or clinical sampling, and the findings should therefore be interpreted as evaluations among the recruited online consumers in China. Online recruitment may also select individuals with particular levels of digital familiarity or willingness to discuss mental health. However, digital literacy and stigma were not directly measured, and the direction of any resulting selection bias cannot be established. Random assignment supports comparisons between the experimental conditions within the recruited samples but does not resolve these representativeness limitations.

The vignette design also limits ecological and clinical inference. Participants evaluated written descriptions and imagined counseling situations rather than receiving counseling or interacting with a deployed LLM. They were not recruited on the basis of a clinically verified acute condition. The findings therefore do not establish actual help-seeking behavior, decision-making during acute distress, or clinical treatment outcomes. Future research should combine more diverse recruitment with studies of actual service interactions and clinically relevant samples under appropriate professional oversight, distinguishing perceptual, behavioral, and clinical outcomes.

Second, several experimental design features limit causal interpretation. The AI counseling system was introduced within a trusted institution with human advisors; although this institutional context was held constant, endorsement information may have shaped perceptions, so the findings apply to institution-backed rather than fully independent AI systems. In Experiment 2, provider-specific attributes—credentials, response efficiency, and pricing—were bundled with provider type rather than independently manipulated. The observed provider type × counseling-need context interaction therefore cannot be attributed solely to clinical severity, and price differences may have contributed to evaluations. In Experiment 3A, the negative news report described an AI-related ethical incident and was presented in both provider conditions. Although this material was constant across conditions, it may have increased the salience of AI-specific risk before the mediator measures. The absence of a parallel human-provider malpractice vignette means the perceived-risk mediation should be interpreted within this primed context. Future studies should orthogonally manipulate provider attributes and use symmetrical provider-specific negative vignettes or a no-prime control condition.

Third, the binary comparison does not represent the full range of collaborative service configurations. In particular, the study did not include a separate condition in which a human professional delivered care with AI assistance while retaining clinical responsibility. The findings therefore cannot establish how consumers would evaluate this configuration or whether it would improve clinical outcomes. Future research should compare AI-based services, human-only services, and AI-assisted human experts, with pricing, response characteristics, and informational content matched where feasible. Such comparisons could examine whether professional oversight changes perceived competence, risk, and efficacy, while evaluating clinical outcomes separately.

## 10. Conclusions

Based on scenario-based experiments with online consumer samples in China, this study draws the following conclusions about perceived efficacy and related evaluations; these conclusions do not establish comparative clinical effectiveness:

(1) Compared with human psychological counseling experts, consumers exhibit lower perceived efficacy of AI-based psychological counseling large language models overall. This main effect is primarily driven by two parallel mediation pathways: lower perceived professional competence and higher perceived risk.
(2) The difference in perceived efficacy between the presented providers varied across counseling-need contexts. Participants rated the AI-based service as more efficacious in the routine counseling vignette, whereas they rated the human-expert service as more efficacious in the major psychological-disorder intervention vignette. These findings reflect subjective expectations under the presented service descriptions, which also differed in provider-specific attributes, and do not establish comparative clinical effectiveness.
(3) Higher perceived economic accessibility was associated with a smaller provider-related difference in perceived professional competence, with evidence of moderated mediation through this pathway. This finding concerns subjective affordability evaluations and does not establish a causal effect of price reductions or differential risk acceptance among income groups.
(4) Proactive disclosure of technological investments by psychological counseling institutions can significantly enhance consumers’ perceived professional competence of AI, whereas disclosure of safety standards can significantly reduce consumers’ perceived risk. Both constitute effective intervention strategies for bridging the human–AI trust gap and enhancing the perceived efficacy of AI-based psychological counseling large language models.

## Data Availability

The original contributions presented in this study are included in the article. Further inquiries can be directed to the corresponding author.
